# Traditional use of ethnomedicinal native plants in the Kingdom of Saudi Arabia

**DOI:** 10.1186/s13002-018-0263-2

**Published:** 2019-01-09

**Authors:** Hanan Aati, Ali El-Gamal, Hamdy Shaheen, Oliver Kayser

**Affiliations:** 1 Department of Pharmacognosy, College of Pharmacy, King Saud University, Riyadh, 11451 Saudi Arabia; 2 Department of Pharmacognosy, Faculty of Pharmacy, Mansoura University, El-Mansoura, 35516 Egypt; 3 Department of English, Faculty of Arts, Mansoura University, El-Mansoura, 35516 Egypt; 4 Technical Biochemistry, TU Dortmund University, Emil-Figge-Strasse 66, 44227 Dortmund, Germany

**Keywords:** Medicinal plants, Saudi Arabia, Ethnomedicine, Traditional use, Folk medicine, Threatened natural resources

## Abstract

The Arabian Peninsula is recognized as an arid area dominated by deserts and poor biodiversity. However, the Kingdom of Saudi Arabia (henceforth abbreviated into KSA) has a wide range of flora, consisting of different species of trees, herbs, and shrubs and containing numerous edible and medicinal plants. The KSA is characterized by its vast area of diverse geographical landscapes and climates. Consequently, there is enormous variation in the distribution of plants across the Kingdom. The traditional use of ethnomedical plants in the KSA represents a strong interconnection among familiar remedies, health, diet, and traditional healing practices characterized by specific cultures.

The present paper reviews a collection of medicinal plants in KSA used in ethnomedicine. This review might be useful in developing strategies for the sustainable use of medicinal plants which are among the threatened important natural resources in folk medicine in the KSA. The present study reports 309 genera which cover 471 species from a total of 2253 known species belonging to 89 families. The most dominating families are Asteraceae, Fabaceae, Lamiaceae, Euphorbiaceae, Solanaceae, Apiaceae, Brassicaceae, Chenopodiaceae, Poaceae, Amaranthaceae, Boraginaceae, Apocynaceae, Convolvoulaceae, Asclepiadaceae, Capparaceae, Polygonaceae, and Zygophyllaceae.

**Electronic supplementary material:**

The online version of this article (10.1186/s13002-018-0263-2) contains supplementary material, which is available to authorized users.

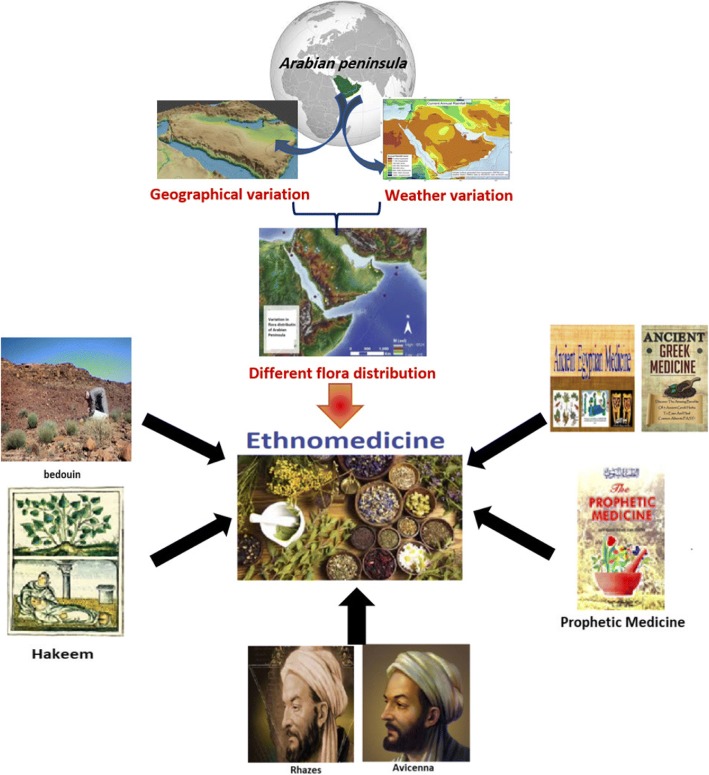

## Background

Plant diversity plays a vital role in serving the ecosystems and in maintaining and preserving ecological balance and stability not only in KSA but also in the whole world as well. Different plant species have been used in ethnomedicine since ancient times [1, 2]. Medicines of the Egyptians (3000 BC; pharaohs), the Greeks (400 BC; Hippocrates), and the Romans (37 BC.; Dioscorides) have a longstanding history. The continuous use of plants in therapy was conducted by Prophet Mohammad (peace be upon him, 571–632 AD); a practice known as The Prophetic Medicine (*Al-Ṭibb al-Nabawi*) by Ibn Qayyim Al-jawziyyah [3]. This period is considered as the golden age for ethnomedicine genesis*.* The Muslims did not stop at that point, but developed different schools, including the Rhazes (865–925 AD) and Avicenna (980–1037 AD), and their encyclopedias on ethnomedicine The Container Book in Medicine (*Kitab Al-Hawi Fi Al-Tibb*) and The Law in Medicine (*Al-Qanun Fi Al-Tibb*), respectively, all of which contributed to the development of herbal medicine [4, 5]. Several medications have been extracted from natural resources, including plants in the nineteenth century. However, The Prophetic Medicine is still a major reference for all Muslims in the Arabian Peninsula and the rest of the world. Many medicinal plants that have been reportedly used in The Prophetic Medicine are currently used in folk medicine in the Arabian Peninsula. Scientific studies have proven that these plants, including garlic, pomegranate, black seeds, costus, miswak, henna, ginger, and fenugreek are effective for treating human diseases. Such plants have been widely used in the form of low cost and almost zero-side-effect products pharmaceutically manufactured and marketed under such trademarks as Black seed plus^®^, Fenugreek 610MG^®^, Ginkago Biloba Plus TM, Kyolic^®^, and Alvita^®^.

**Fig. 1:**
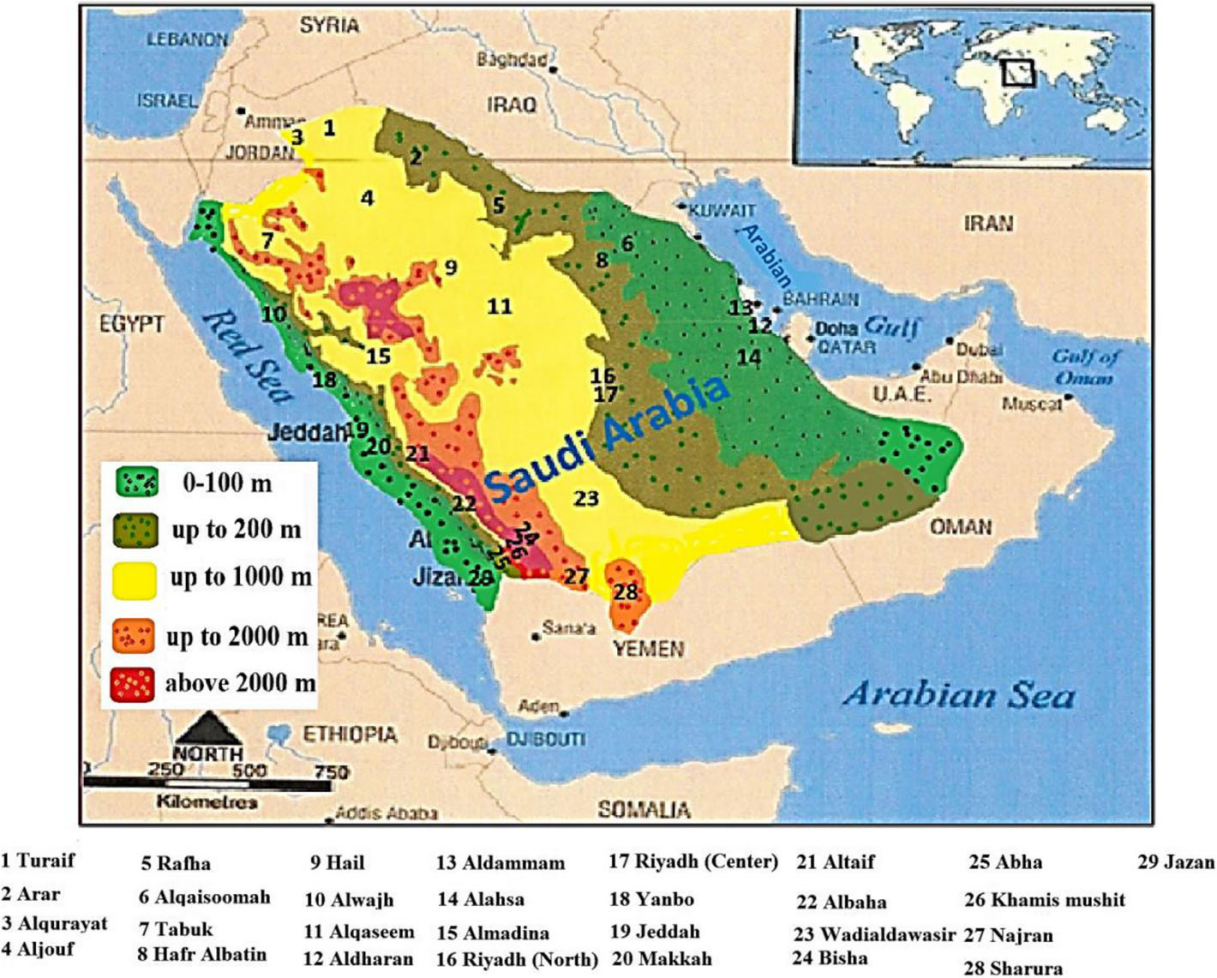
Geographical areas of Saudi Arabia

The KSA, Kuwait, Bahrain, Yemen, Qatar, United Arab Emirates, and Oman formulate what is known as the Arabian Peninsula which is located in the Asian southwest. Its West and Southwest border is the Red Sea, its southern one is the Gulf of Aden, its southern and southeastern border is the Arabian Sea, and its eastern one is the Gulf of Oman and the Arabian Gulf [6]. Figure 1 highlights the geographical merge of the peninsula with the Syrian desert across the northern border, but the northern boundaries of Saudi Arabia and of Kuwait are generally considered as marking the limit of Arabia there.

The 2,250,000 km^2^ covered by KSA represent the largest part of the Arab Peninsula. Geographically, it is characterized by a variety of habitats including mountains, valleys, lava areas, meadows, and rocky deserts. Therefore, KSA is made up of two areas: the rain fed zones of the western and southwestern highlands and the arid region of the interior area [6].

As for the first division, the Asir highlands constitute a flowing series of cliffs together with similar southwestern highlands, extending far unto the Yemeni borders. They rise up to 2000 m in some areas (Taif) and over 3000 m in others (Abha). The same continuity is to be found in the western continuous chain across the Tihama coastal plain. This continuity comes to an end on the northwestern sides where discontinuity dominates. On the other hand, the eastern part comprises a large amount of sand with much lower mountains and plains. These deserts extend to the northern Great Nafud and the southern Rub AlKhali (The Empty Quarter), with Dahna sands connecting both regions. As regards the Central Region, patchy, stone deserts, hillocks, and valleys color the scene versus the complex of metamorphic rocks that characterize the northern and northern central region. Remarkable still is that the altitudes of the plains of the Central Region rise high up to 500–800 m as compared to such mountains and escarpments Jabal Shammar and Jabal Tuwayq which range from 300 to 600 m [6]. Forests cover 2.7 million hectares (about 1.35% of the total area) of KSA. Most of the forests are in the southwestern part of the Kingdom on the Sarawat mountain.

With regard to the location of KSA, it is located in a climatic transitional zone of tropical and temperate climates. Moreover, the rains falling upon KSA are neither constant in amount and nor equal in distribution so much that the arid areas right in the interior of KSA underscore no rain in some areas and over 200 mm in some others on an annual basis. Even in winter, there is rainfall, however irregular.

It is noteworthy that winter in KSA ranges from cool to warm (2–20 °C) as opposed to the high summer temperature ranging from 35 to 50 °C, high humidity along the Red Sea coast and the Arabian Gulf coast; temperature becomes lower right in the interior.

Geographical and climatic diversity in the kingdom lead to the diversity in the flora, examined for the first time in 1974 [7]. This was followed by two volumes of Medicinal Plants of Saudi Arabia that were published in 1987 and 2000 by Mossa et al. [8, 9].

Collenette (1998) asserts that KSA produces about 837 genera manifesting themselves in 2253 species distributed among 132 families. Nearly 20% of these families stands for totally new and uncommon plants. The southwestern part of KSA embraces about 70% of the floristic elements grown across Taif to the Yemeni border. This is accounted for the regularity of rainfall upon the region [10].

Equally important is KSA flora provide a remarkably rich source for agriculture and medicinal plants. Although many items of the flora are grown locally and nationally, KSA still grows a curious amalgam of Africa, Asia, and the Mediterranean region.

According to Al-Yahya (1984), employing and resorting to traditional medicine is still a fashionable practice nowadays in KSA [11]. It has become customary for KSA citizens to resort to natural herbs and traditional remedies in the hope that these will certainly heal their illness. Such practices are found in using such natural resources as bee-honey, black seed, myrrh, fenugreek, kawajawa, and among others. It has been to their good fortune that these practices have been handed from one generation to another. Mossa et al. (1987) spotlight the extensive range of flora that covers almost the whole KSA and offers sample opportunity for Saudis to make the best use of herbs and flora for their medications to local individuals for use in and therapeutic practices [8, 9].

The KSA is currently implementing an overall plan with a view to regulating and organizing the use of traditional medicine on a national basis. Thus, there is a tendency to legalize what is known as Complementary and Alternative Medicine (TM/CAM) via issuing organizing acts and issuing laws. This has been consolidated by the establishment of the National Office in 1995 on behalf of the Saudi Ministry of Health. Another important step came with the TM/CAM Committee in collaboration with the National Research Institute on herbal medicine studies established by King Saud University Medicinal, Aromatic and Poisonous Plant Research Center (MAPPRC), and the Department of Pharmacognosy, both of the College of Pharmacy.

Historically, these practices in KSA date back to ancient times when using traditional medicine was the only way out for the treatment of many illnesses and diseases that were usually unknown and non-diagnosed. However, these medicines were too much respected as they were prescribed by those traditional healers known as Al-Hokama. It is worthy of notice, however, that such practices are beginning to vanish and pass away. That is why it has become a prerequisite to conduct folk and ethnobotanical surveys and studies in KSA on the vanishing of these practices before they become history. As result, this review can serve as a reference document on traditional uses of medicinal plants in Saudi Arabia and increase the possibility of discovering new drug resources.

## Materials and methods

Journals, textbooks, proceedings, websites, periodicals, and databases dealing with medicinal plants used to treat human diseases in Saudi Arabia, Arabian Peninsula, and other parts of the world were checked for related information. Dictionaries of English/Arabic and Arabic/English were also consulted for accuracy.

## Results and discussion

**Fig. 2:**
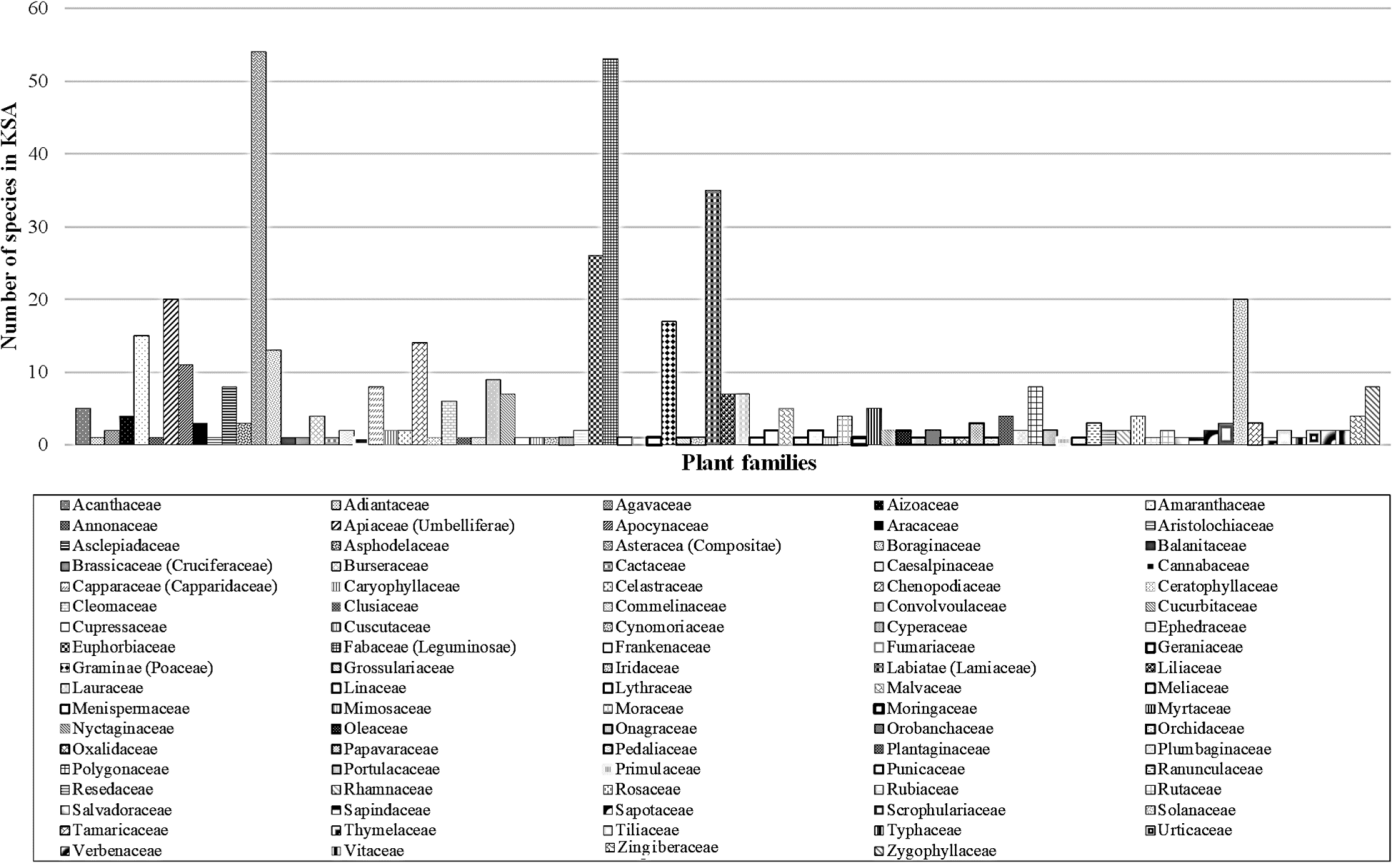
Number of traditionally used species per family in Saudi Arabia

Literature survey showed that a total of 309 genera containing 471 species in 89 families are used in ethnomedicine (Table 1 and Fig. 2). Asteraceae and Fabaceae families have the highest number of ethnomedicinal species (54 and 49, respectively) in Saudi Arabia. Additional file 1: Table S1 summarizes ethnobotanical data on all medicinal plants used in KSA [12–41].

**Table 1:** Number of traditionally used species per family in Saudi Arabia

Family name	Number of species in KSA	Family name	Number of species in KSA
Acanthaceae	5	Lauraceae	1
Adiantaceae	1	Linaceae	1
Agavaceae	2	Lythraceae	2
Aizoaceae	4	Malvaceae	5
Amaranthaceae	13	Meliaceae	1
Annonaceae	1	Menispermaceae	2
Apiaceae (Umbelliferae)	18	Mimosaceae	1
Apocynaceae	10	Moraceae	4
Aracaceae	3	Moringaceae	1
Aristolochiaceae	1	Myrtaceae	5
Asclepiadaceae	9	Nyctaginaceae	2
Asphodelaceae	4	Oleaceae	2
Asteracea (Compositae)	54	Onagraceae	1
Boraginaceae	13	Orobanchaceae	2
Balanitaceae	1	Orchidaceae	1
Brassicaceae (Cruciferaceae)	17	Oxalidaceae	1
Burseraceae	5	Papavaraceae	3
Cactaceae	1	Pedaliaceae	1
Caesalpinaceae	1	Plantaginaceae	4
Cannabaceae	1	Plumbaginaceae	2
Capparaceae (Capparidaceae)	8	Polygonaceae	8
Caryophyllaceae	2	Portulacaceae	2
Celastraceae	2	Primulaceae	1
Chenopodiaceae	15	Punicaceae	1
Ceratophyllaceae	1	Ranunculaceae	3
Cleomaceae	6	Resedaceae	3
Clusiaceae	1	Rhamnaceae	2
Commelinaceae	1	Rosaceae	4
Convolvoulaceae	9	Rubiaceae	1
Cucurbitaceae	6	Rutaceae	2
Cupressaceae	2	Salvadoraceae	1
Cuscutaceae	1	Sapindaceae	1
Cynomoriaceae	1	Sapotaceae	2
Cyperaceae	2	Scrophulariaceae	2
Ephedraceae	1	Solanaceae	20
Euphorbiaceae	26	Tamaricaceae	3
Fabaceae (Leguminosae)	49	Thymelaceae	1
Frankenaceae	1	Tiliaceae	2
Fumariaceae	1	Typhaceae	1
Geraniaceae	1	Urticaceae	2
Graminae (Poaceae)	13	Verbenaceae	2
Grossulariaceae	1	Vitaceae	2
Iridaceae	1	Zingiberaceae	4
Labiatae (Lamiaceae)	33	Zygophyllaceae	7
Liliaceae	4

Table 1 shows numerous plant species used for various illnesses associated with gastrointestinal problems, pains, rheumatism inflammations, ulcers, respiratory, circulatory, urological, and skin diseases, and some for toothache, diabetes, allergy, and gynecology. The most mentioned medicinal plant families were Asteraceae, Fabaceae, Lamiaceae, Euphorbiaceae, Solanaceae, Apiaceae, Brassicaceae, Chenopodiaceae, Poaceae, Amaranthaceae, Boraginaceae, Apocynaceae, Convolvoulaceae, Asclepiadaceae, Capparaceae, Polygonaceae, and Zygophyllaceae. All these families as well as other families mentioned in this review are already represented in Saudi Arabia flora [7, 42].

Resorting to traditional medicinal plants for treating some illnesses has resulted in many the production of many promising drugs and medications [43, 44]. The present paper reviews these practices from an ethnopharmacological perspective by targeting 471 medicinal plants used on a regular basis by almost all Saudis (Additional file 1: Table S1).

According to Ali et al. (2017), these therapeutic practices and use of medicinal plants appear on the Use Index (UI), [UI = (na/NA × 100), where na is the number of questioners who refer to the species as valuable and NA is the total number of people met]. Three vital plants were at the core of Saudis’ practices: *Juniperus procera*, *Rumex nervosus*, and *Ziziphus spina-chris* [33]. Therefore, these three important and commonly used plants have been discussed here in detail.

*Juniperus procera* Hochst. ex Endl is known as “Arar” in KSA. It is a long tree that reaches up to 8 m tall with needle-shaped leaves, extending from the south reigon of the Arab Peninsula across the Red Sea into Africa [45]. *Juniperus* is one of the major genera of Cupressaceae family consisting of approximately 70 species [46]. Traditionally in Saudi Arabia, *J. procera* used for treating hepatic diseases, jaundice, gastrointestinal disturbances, and pharyngitis, as antirheumatism, for gout and several inflammatory conditions [21, 47]. In ethnomedicine, the resinous material of *J. procera* was added to bee-honey and used as a remedy for curing hepatic and skin diseases [48].

A previous phytochemical study of different parts of *J. procera* resulted in the isolation of different classes of sesquiterpenes and diterpenes, where the stem bark of *J. procera* afforded two lignans identified as β-peltatin and deoxypodophyllotoxin [95]. In addition, the stem barks and leaves of the same plant gave two diterpenes designated as totarol and sugiol with strong antimicrobial activity [49, 50]. Abietane, pimarane, and labdane types of diterpenes have been isolated from *J. procera* fruits which showed antiparasitic and nematicidal activities [51]. Moreover, totarol exhibited a synergistic effect with isoniazide (INH) towards four *Mycobacterium* species [52]. In addition, its essential oil possesses significant antioxidant/free radical scavenging activity [53].

The less polar fraction of the aerial part of *J. procera* exhibited significant hepatoprotective effect against liver toxicity induced by CCl_4_. The hepatoprotective activity has been referred to the presence of terpenes in good yield: 4-*epi*-abietol, ferruginol, hinokiol, sugiol, *Z*-communic acid and hinokiol-1-one, and 3-*β*-12-dihydroxyabieta-8,11,13-triene-1-one, in addition to 8-*α*-acetoxyelemol sesquiterpene [54]. A toxicity study of *J. procera* extract revealed the safety of the extract even at high doses with no acute or chronic toxicity appearance [48]. Additional file 1: Figure S1 shows the structures of the major compounds isolated from *J. procera*.

*Rumex nervosus* Vahl. is known locally as “Ithrib.” Some people in the Arabian Peninsula especially in Yemen and Saudi Arabia consume *R. nervosus*, which makes it an edible plant. However, if eaten in large quantities, it can produce toxic effects because of its high calcium oxalate content [55]. *Rumex nervosus* belongs to the family Polygonaceae and is a large annual herb that can grow up to 1.5 m tall, its leaves are usually sagittate, the inflorescence is considerably branched, and it has a leafless panicle. It has a light brown nut, and its fruits are cordate-orbicular. It is widely distributed in Yemen, Saudi Arabia, Ethiopia, Somalia, Kenya, and Tanzania. Ithrib is a very common plant that is used by native people as a diuretic, antipyretic, and antirheumatic and to treat gonorrhea, leprosy, lung tuberculosis, and liver illness; as antihypertensive, antihemorrhoids, antiscabies, antiemetic, aphrodisiac, antitussive, and antirabies; and for dermatitis, antiacne, hypoglycemic, antiinfective, and headache. Decoction of the leaf or root powder produces a substance that is used as a vermifuge. Moreover, the leaves of *R. nervosus* are utilized to treat skin rashes and young leaves are roasted to decrease the acid content before being eaten. The material formed after the burning of the stem is mixed with egg yolk or butter and applied to burns [55–59].

There are some reports on the anthelmintic [55], anti-inflammatory and antiviral [57], analgesic [58], diuretic and laxative [59], and antioxidant [60] effects of *R. nervosus*.

It has been confirmed that *R. nervosus* extracts contain alkaloids, flavonoids, phenols, amino acids, furanocoumarins, and saponins by preliminary phytochemical screening [61].

Moreover, the genus *Rumex* is characterized by the presence of anthraquinones, naphthalene-1,8-diols, flavonoids, and stilbenoids [62]. Many phenolic compounds have been isolated from the *R. nervosus* ethyl acetate fraction, including kaempferol, quercetin, gallic acid, hyperoside, quercetin 3-*O*-(6″-acetyl)-galactoside, hesperidin, quercetin, *p*-hydroxy benzoic acid, catechol, and pyrogallol [63]. Additional file 1: Figure S2 shows the structures of the major compounds isolated from *R. nervosus*.

*Ziziphus spina-christi* is commonly called “Jujube” and is locally known as “Sidr” or “Nabuk.” It is a multiuse tree belonging to the family Rhamnaceae. Sidr is a native plant that grows in tropical and subtropical regions, and it is widely distributed throughout the Mediterranean regions, Africa, Asia, and tropical America. In Saudi Arabia, Sidr is wildly distributed throughout the southern and southwestern area and has been used as an ornamental plant and for shade. It is a tall tree that can reach 20 m in height. Its leaves are glabrous on the upper surface, finely pubescent on the lower surface, and ellipsoid or ovate lanceolate in shape with an obtuse or acute apex [64].

*Ziziphus spina-christi* is greatly respected by Muslims since it is mentioned in the Sunnah and the Holy Quraan twice [65]. From ancient times, in Chinese traditional medicine, suan zao ren (*Z. spinosa*) has been used to increase blood flow to the heart and liver, and it is used to control irritability, insomnia, and palpitations [66]. In Saudi folk medicine, the leaves of *Z. spina-christi* (jujube) are used to heal wounds, treat some skin diseases and sores, cure ringworm, antipyretic, gonorrhea, sex diseases, some inflammatory conditions, and ulcers. Furthermore, it has been reported that *Z. spina-christi* leaves are used in folk medicine as antidiabetic remedy [67]. In the Bedouin, the decoction of the stem bark and fresh fruits is used as a body rinse, to cure fresh wounds, and is also used for the management of dysentery, bronchitis, coughs, and tuberculosis [68].

Previous phytochemical investigation revealed it contains biologically active secondary metabolites including, tannins, flavonoids, terpernoids, saponin glycosides, and alkaloids [68].

Pharmacological studies have demonstrated that the total alcoholic extract of the current plant leaves and stem bark has showed significant antioxidant, antimicrobial, and antidiarrheal activities [66, 69]. Additionally, the aqueous extract from the root bark has an antinociceptive activity [70] and central nervous system depressant effect in mice [71]. The butanol extract of *Z. spina-christi* leaves has shown potent hypoglycemic/antidiabetic activities [72]. The aqueous and ethanolic extracts of stem bark of *Z. spina-christi* have been studied, and an anticholinergic effect was observed [73], which proved the traditional use of the plant as antispasmodic. A cytotoxic effect was observed for the aerial part of *Z. spina-christi* against cervical, breast, and colon cancers [66].

A phytochemical study of *Z. spina-christi* indicated the presence of betulic and ceanothic acids [74]. Cyclic peptide alkaloids, franaganine, mauritine C, and sativanine A have been isolated and fully characterized from the stem bark of *Z. spina-christi* [75]. Triterpenoidal saponins were recently isolated from the leaves of the same plant and screened for their antidiabetic activity [76]. Additionally, four saponin glucosides identified as christinin A–D have been isolated from the leaves *n*. butanol extract leaves and their structures were fully characterized by using spectroscopic technique [77]. Furthermore, quercetin, hyperoside, ruin, and quercetin-3-*O*-[*β*-xylose-(1–2)-*α*-rhamnose] 4′-*O*-*α*-rhamnose have also been isolated from the leaves and fruits of *Z. spina-christi* [78]. Additional file 1: Figure S3 shows the structures of the major compounds isolated from *Z. spina-christi*.

## Conclusions

Ethnomedicinal knowledge is not transferred from the older generations to the young age generations, meaning it will soon be erased especially as most younger individuals prefer to visit clinics and hospitals more regular than older people and the Bedouin. Moreover, folkloric healers (Hakeem) use wild herbs randomly and without any restriction, which increases the chance of extinction of certain medicinal plants. This requires greater consideration because more than 15,000 plant species might face extinction worldwide due to over-harvesting and misuse [2, 14]. Therefore, owing to the high diversity of medicinal plants in the KSA, the present review recommends the following:

- Phytochemical and pharmacological studies on the different flora plants.
- Creation of distribution maps for important medicinal plants using GPS coordinates.
- Data analysis of medicinal plants based on their phytochemical, chemotaxonomical, and pharmacognestical characteristics.
- Monographs of some pharmacopoeial medicinal plants.
- Continuing the surveying of plants and identification of their exact locations.
- Plants provided with sequence bank and fingerprinting.
- Seed library for the plants or seed bank to preserve genetic diversity and to preserve rare plant species.

A total of 471 plant species belonging to 89 families were recorded in the present review from the KSA, which means that this country has a large number of medicinal plants that need to be discovered and have their chemical and pharmacological properties studied.

The findings from the present review paper lend support to the extensive traditional medicinal knowledge in the KSA, which provide the basis for further medicinal research on a scientific evidence basis.

The current study has reviewed a total of 471 plant species belonging to 89 families grown in KSA. This highlights the vast wealth of medicinal plants in KSA. These plants have been used as medications for healing and treating many diseases on an ethnomedicinal, folk medicinal, and national basis. Practicing traditional medicine by resorting to these plants in KSA dates back to ancient times so that it has become fashionable among almost all Saudis.

Therefore, it is suggested that further studies be conducted on unveiling the pharmaceutical, pharmacological, chemical, and laboratory properties of these medicinal plants that have never been targeted before via studies from this perspective. Thus, this tremendous treasure of medicinal plants in KSA needs a greater deal of attention so that researcher would direct future studies to dig out the undiscovered secrets of these plants from a scientific angle of vision. It is hoped that these future studies, once scientifically conducted, will come up with new discoveries in the form of new drug leads extracted from natural resources that would utterly changes the medication shelves all over the world.

## Additional file

Additional file 1: **Table S1.** Ethnobotanical information on some medicinal herbs used in different regions of Saudi Arabia. **Figure S1.** Structures of the major compounds isolated from *J. procera.* **Figure S2.** Structures of the major compounds isolated from *R. nervosus.* **Figure S3.** Structures of the major compounds isolated from *Z. spina-christi*. (DOCX 771 kb)

## Abbreviations

KSA: Kingdom of Saudi Arabia
MAPPRC: Medicinal, Aromatic and Poisonous Plant Research Center
TC/CAM: Traditional medicine and/or complementary medicine
UI: Use index
