# Supplementary material for: Traditional use of ethnomedicinal native plants in the Kingdom of Saudi Arabia
Source: J Ethnobiol Ethnomed. 2019 Jan 9;15:2. doi: 10.1186/s13002-018-0263-2 (PMC6325684; doi:10.1186/s13002-018-0263-2)
Supplement: Supplementary file 1 — Table S1. Ethnobotanical information on some medicinal herbs used in different regions of Saudi Arabia. Figure S1. Structures of the major compounds isolated from J. procera. Figure S2. Structures of the major compounds isolated from R. nervosus. Figure S3. Structures of the major compounds isolated from Z. spina-christi. (DOCX 771 kb) [file 13002_2018_263_MOESM1_ESM.docx]

**Supplementary Material to**

**Traditional Use of Ethnomedicinal Native Plants in The Kingdom of Saudi Arabia**

Hanan Aati^1^, Ali El-Gamal^1,2^, Hamdy Shaheen^3^ and Oliver Kayser^4^

^1^Department of Pharmacognosy, College of Pharmacy, King Saud University, Riyadh 11451, Saudi Arabia, hati@ksu.edu.sa

^2^Department of Pharmacognosy, Faculty of Pharmacy, Mansoura University, El-Mansoura 35516, Egypt, aelgamel@ksu.edu.sa

^3^Department of English, Faculty of Arts, Mansoura University, El-Mansoura 35516, Egypt, [hamdiishaheen@gmail.com](mailto:hamdiishaheen@gmail.com)

^4^TU Dortmund University, Technical Biochemistry, Emil-Figge-Strasse 66, D-44227 Dortmund, Germany, oliver.kayser@tu-dortmund.de

Corresponding Author:

Oliver Kayser

TU Dortmund University

Emil-Figge-Strasse 66

44227 Dortmund

Germany

Tel: +49 231 7557487

E-Mail: [oliver.kayser@tu-dortmund.de](mailto:oliver.kayser@tu-dortmund.de)

| Table S1: Ethnobotanical information on some medicinal herbs used in different regions of Saudi Arabia | | | | | | |
| --- | --- | --- | --- | --- | --- | --- |
| **Scientific name** | **Local name** | **Family** | **Part used** | **Traditional uses** | **Region** | **Reference** |
| *Blepharons ciliaris* L. | Shook aldabb | Acanthaceae | Leaves & whole plant | Toothache & skin wound. | M, W, S | 12, 13, 14, 15, 16 |
| *Blepharis maderaspatensis* | Athrarh |  | Leaves | Used as treatment for scorpion sting. | - | 17 |
| *Ecbolium viride* (Forssk) | Khoseer |  | Leaves powder | Treat pimples & when applied around genital area it improves urination. | S | 15, 16 |
| *Ecbolium gymnostachyum (Nees)* | Syhan |  | Leaves | Wound healing & bone fracture. | M | 13 |
| *Hypoestes forsskalii* (Vahl) | Nadqah |  | Leaves | Anti-inflammatory & wound healing in cattle. | M | 13 |
| *Adiantum capillus-veneris* | Kizbratal Baer | Adiantaceae | Whole plant | Antipyretic, antitussive, diuretic, emmenagogue, expectorant & pulmonary catarrh. | M, W | 12, 13 |
| *Dracaena ombet* Kotschy & Peyr. | Azef | Agavaceae | Resin | Used as antihemorrhagic & for skin infections. | S | 15, 18 |
| *Sansevieria ehrenbergii* Schweinf. ex Baker | Salaf |  | Leaves | Used for treating blisters. | S | 15, 18 |
| *Aizoon canariense* L. | Hodaq | Aizoaceae | Whole plant | Used to treat indigestion flatulence & hypertension. | M | 13 |
| *Mesembryanthemum crystallinum* | Nabat Al-thalg |  | Whole plant | Antimicrobial. | S | 19 |
| *Mesembryanthemum forsskalei* | Samh |  | Seeds | Making bread. | S | 19 |
| *Trianthema portulacastrum* | Laani |  | leaves | Used as treatment for scorpion sting. | - | 17 |
| *Achyranthes aspera* L. | Mahwat | Amaranthaceae | Leaves, roots, seeds & barks | Stomachache, bowel complaints piles, boils, scorpion sting & skin eruptions. | S, W | 15, 17 |
| *Aerva javanica* (Bunn.f.) | Tuwain |  | Whole plant | Toothache, hemorrhage, snake & insect bites. | M, W, S | 12, 13, 15 |
|  |  |  | Roots (juice) | Treat eye diseases in cattle. |  |  |
| *Aerva lanata* L*.* | Al-Athlab |  | Whole plant | Diuretic, scorpion sting & demulcent. | M, S | 13, 15, 8, 17 |
| *Alternanthera pungens* Kunth. | - |  | Whole plant | Used as treatment for scorpion sting. | M | 17 |

| Table S1: Cont. | | | | | | |
| --- | --- | --- | --- | --- | --- | --- |
| **Scientific name** | **Local name** | **Family** | **Part used** | **Traditional uses** | **Region** | **Reference** |
| *Alternanthera sessilis* L. R.Br. ex DC. | - | Amaranthaceae | Leaves | Used as treatment for scorpion sting. | M | 17 |
| *Amaranthus caudatus L.* | Kaf-Almehana |  | Whole plant | Diuretic, strangury, blood purifier & for the treatment of piles. | S | 15 |
|  |  |  | Leaves | Used as an abortifacient. |  |  |
| *Amaranthus graecizans* L. | Thaflah |  | Leaves | Chewed & the liquid swallowed to treat tonsillitis, scorpion sting & used as anthelmintic. | M | 13, 17 |
| *Amaranthus hybridus* Spinach | Sabanih |  | Whole plant | Treat jaundice, inflammation, blood tonic, laxative & as digestive. | - | 20 |
| *Amaranthus retrofexus* | Oshba alqanzeer |  | Leaves | Astringent & anthemorrhagic. | M | 13 |
| *Amaranthus spinosus* L. | Da^,^ad |  | Whole plant | Antipyretic, diuretic, laxative, scorpion sting & stomachic. | S | 15, 17 |
|  |  |  | Roots | Treat gonorrhea & constipation & jaundice. |  |  |
| *Amaranthus viridis* L. | Shae |  | Whole plant | Antipyretic, diuretic, emollient in scorpion sting, expectorant, laxative & leprosy | M, S | 13, 15, 17 |
| *Bassia muricata* L. | Hassaniya |  | Whole plant | Antimicrobial. | S | 15, 19 |
| *Celosia trigyna* L. | Trgana |  | Leaves & flowers | Treatment of diarrhoea & for excessive menstruation | S | 13, 19 |
| *Annona squamosa* L. | - | Annonaceae | Leaves, roots & barks | Used as treatment for scorpion sting. | - | 17 |
| *Ammi majus* | - | Apiaceae  (Umbelliferae) | Whole plant | Skin diseases, leukoderma, contraceptive, diuretic, tonic, angina, carminative, antiasthmatic & toothache. | M | 12 |

| Table S1: Cont. | | | | | | |
| --- | --- | --- | --- | --- | --- | --- |
| **Scientific name** | **Local name** | **Family** | **Part used** | **Traditional uses** | **Region** | **Reference** |
| *Anethum graveolens* L. | Dill, Shibt | Apiaceae  (Umbelliferae) | Whole plant, roots, leaves, seeds, fruits | Antimycobactetial, Antifungal, insecticides, psychoactive, hallucinogenic, appetizer, colic gripes, mouth wash, carminative, aphrodisiac, diuretic, astringent, cordial, laxative, stimulant, emmenagogue, anthelmintic, abortifacient, antispasmodic, treating bronchitis, liver and spleen disorder, lumbago & galactagogue. | S, M, W | 12, 15, 13 |
| *Apium graveolens* | Karfas |  | Flowers  roots & seeds | Digestive, antacid, body tonic, laxative, chest ailments, arthritis, jaundice, appetizer, colic gripes, tonic, purgative, vermifuge, antiasthma, liver and spleen disorder, antirheumatic, anasarca, homeopathic, antispasmodic, sedative, high blood pressure, kidney problems, anticonvulsant & calming. | M | 12, 20 |
| *Bepleurum semicompositum* | - |  | Fruits | Stomach troubles, carminative & mentalis disorders. | M | 12 |
| *Carum carvi* L*.* | - |  | Roots | Used as treatment for scorpion sting. | - | 17 |
| *Conium maculatum* L. | Shawkaran |  | Flowers & leaves | Used as treatment for scorpion sting. | - | 17 |
| *Coriandrum sativum* L. | Kosbarah |  | Whole plant | Antiflatulence, antidiarrhea, anticolic & as general tonic. | S | 15 |
| *Cuminum cyminum* | Kamun |  | Seeds & leaves | Diuretic, carminative, anticolic, antioxidant, uterine discharge, hiccup, appetizer, deafness, spasm & digestive. | S | 21, 20 |
| Table S1: Cont. | | | | | | |
| **Scientific name** | **Local name** | **Family** | **Part used** | **Traditional uses** | **Region** | **Reference** |
| *Daucus carota* Linn*.* | Jizr | Apiaceae  (Umbelliferae) | Leaves & roots | Mucolytic, chest pain, cough, hepatic, gastric problems, diuretic, brain stimulant, coarseness of voice, digestive, fatigue, hypertension, nervousness & skin diseases. | - | 20 |
| *Ducrosia ismaelis* L*.* | - |  | Whole plant | Antimicrobial. | S | 15, 19 |
| *Echinosciadium arabicum* L. | - |  | Whole plant | Central nervous system stimulation & antimicrobial. | S | 15, 19 |
| *Eryngium foetidum* L*.* | Cilantro |  | Whole plant | Used as antipyretic, antiemetic & antidiarrheal. | S | 15, 18 |
| *Foeniculum vulgare* Mill. | Shawmar,  Sheeh |  | Whole plant  seeds, leaves & stems | Used to relieves digestive problems, increases lactation, antiflatulence, reduces inflammation, antimicrobial, used for skin disorders, antitussive, stomach problems, as toothbrush, used for conjunctivitis & used for blepharitis of the eye. | W | 13, 22 |
|  |  |  |  |  | S | 15, 16, 23 |
| *Petroselinum crispum* L. | - |  | Whole plant | Used against kidney stones. | S | 15 |
| *Petroselinum sativum* L. | Maqdunes |  | Whole plant & root | Used against kidney stones, diuretic & liver disease. | S | 15, 23, 20 |
| *Pimpinella anisum* Lnn. | Yanisun |  | Leaves & seeds | Purgative, cough, mucolytic, headache, dejection & earache. | - | 20 |
| *Pituranthus triradiatus* L. | - |  | Whole plant | Treat hypertension &as antimicrobial. | S | 15, 19 |
| *Trachyspermum ammi* L. | Kemyon, Nahwa |  | Seeds & stem | Used against kidney stones & used as treatment for scorpion sting. | S | 15, 23 |
| *Adenium arabicum* Balf. | Adnah | [Apocynaceae](http://www.theplantlist.org/1.1/browse/A/Apocynaceae/) | Barks & whole plant | Used in bones dislocations, painful joints, wounds & skin infections. | S | 15, 21 |
| *Adenium obesum* L. | Aden |  | Whole plant & barks | Applied on wounds as antimicrobial & used as treatment for scorpion sting. | S | 15, 16, 19, 17 |
| *Blyttia reticuloses* L. | - |  | Whole plant | Antimicrobial. | S | 15, 19 |

| Table S1: Cont. | | | | | | |
| --- | --- | --- | --- | --- | --- | --- |
| **Scientific name** | **Local name** | **Family** | **Part used** | **Traditional uses** | **Region** | **Reference** |
| *Canthranthus roseus* | Biftah | [Apocynaceae](http://www.theplantlist.org/1.1/browse/A/Apocynaceae/) | Leaves & roots | Digestive, astringent, emetic, purgative, hemorrhagic, emmenagogue, diabetes, used as treatment for scorpion sting & cancer. | M | 12, 17 |
| *Carlumab penicillata* (Defl.) | Ghaltha |  | Whole plant & leaves | Central nervous system stimulation, used in diabetes, stomach ulcer & smallpox. | S | 15 |
| *Carissa edulis* Vahl Symb. Bot. | A'rm, Airoon |  | Whole plant | Anthelmintic, antiscorbutic, astringent, stomachic & toothache. | S | 15 |
| *Holarrhena pubescens* (Buch.-Ham.) | Kurchi |  | Seeds & barks | Treating painful joints & in diarrhea. | S | 21 |
| *Nerium oleander* L*.* | Dafla |  | Leaves & roots | Treat skin disease & steam of boiled leaves inhaled in sinusitis. | W, S | 15, 16 |
| *Rhazya stricata* Decne. | Harmal |  | Whole plant | Antirheumatic, pain relief, antibacterial & treatment for allergy. | M, S | 12, 14, 15, 17 |
| *Torularia torulosa* L*.* | - |  | Whole plant | Lower blood pressure & cardiac stimulation. | S | 15, 19 |
| *Arisaema flavum* | Dakaf | Aracaceae | Rhizome | Used as treatment for scorpion sting. | - | 17 |
| *Hyphaene thebaica* L*.* Mart., Hist. | Doom |  | Whole plant | Cardiac stimulation & antimicrobial. | S | 15, 19 |
| *Phoenix dactylifera* L. | Nakhl |  | Flower & pollen | Used as an aphrodisiac & as a general tonic. | S, W, M | 21 |
| *Aristolochia bracteolata* Lam. | Loiya | Aristolochiaceae | Leaves | Used to treat snake bite & scorpion sting. | S | 16, 17 |
| *Calotropis procera* Ait. | Oshar | Asclepiadaceae | Whole plant  & latex | Respiratory system, antiasthmatic, anticancer, joint inflammation, antibacterial, purgative, expectorant, dysentery, emetic & for leshmaniasis. | M, W, S | 12, 13, 14, 16 |

| Table S1: Cont. | | | | | | | | | | | | | |
| --- | --- | --- | --- | --- | --- | --- | --- | --- | --- | --- | --- | --- | --- |
| **Scientific name** | | **Local name** | | **Family** | | **Part used** | | **Traditional uses** | | **Region** | | **Reference** | |
| *Caralluma sinaica* (Decne.) | | Ded-Elkalba | | Asclepiadaceae | | Whole plant & leaves | | Hypoglycemic & antiprotozoal. | | S | | 15, 24 | |
| *Caralluma tuberculata* N. E. Br. | | Pawany | |  |  | Whole plant | | Used in the case of diabetes, peptic ulcers, inflammation & its juice as drops for ear inflammation. | | S, W | | 16 | |
| *Cynanchum acutum* L*.* | | Al-Modeed | |  |  | Leaves & stems | | Insecticide & parasiticide. | | S | | 15 | |
| *Gomphocarpus fruticosus* L. Ait.f. | | Alqaraa Kabeer | |  |  | Leaves | | Tumors, skin disease, scabies & itching. | | S | | 25 | |
| *Gomphocarpus sinaicus* | | Arjal | |  |  | Whole plant | | Used for hemorrhagic disorders, rhinorrhagia & metrorrhagia. | | M | | 12 | |
| *Leptadenia pyrotechnica* (Forssk.) | | Markh | |  |  | Whole plant & seeds | | Used for flu, tussive, lactagogue & antimicrobial. | | M, S | | 12, 15 | |
| *Pergularia tomentosa* L. | | Gholfa | |  |  | Whole plant | | Skin diseases. | | M, W, S | | 13, 14, 15 | |
| *Periploca aphylla* L*.* | | Sawas | |  |  | Whole plant | | Antiprotozoal, stomachic & purgative. | | S | | 18, 24 | |
| *Aloe tomentosa* Defl. | | Sabbar | | Asphodelaceae | | Leaves & sap | | Applied on skin for rashes & sunburn. Sap is applied for inflammation of eyes. | | S | | 16, 21 | |
| *Aloe vera (L.) Burm. f.* | | Saqal, Sabar | |  |  | Whole plant, Juice of leaves, bulbs & roots | | As laxative, in asthma, peptic ulcers, burns & diabetes. | | S | | 15, 26 | |
| *Asphodelus centifolias* L. | | Broque | |  |  | Seeds | | Used in colds, haemorrhoids & as antirheumatic. | | S | | 15 | |
| *Asphodelus fistulosus* | | Towaim, | |  |  | Whole plant & seeds | | Diuretic, ulcers, toothache & antiinflammatory. | | W, M, S | | 12, 14, 15 | |
| *Achillea biebersteinii* Afan. | | Thafraa | | Asteraceae  (Compositae) | | Leaves | | Strong antimicrobial activity, relieve itching, remove readiness & swelling in eyes. | | S | | 15, 19, 21 | |
| Table S1: Cont. | | | | | | | | | | | | | |
| **Scientific name** | | **Local name** | | **Family** | | **Part used** | | **Traditional uses** | | **Region** | | **Reference** | |
| *Achillea fragrantissima* L. | | Qusom | | Asteraceae  (Compositae) | | Whole plant | | Central nervous system stimulation & antimicrobial. | | S, M | | 15, 19 | |
| *Ambrosia maritima* L. | | Ambrosia | |  |  | Whole plant | | Antiflatulence & diuretic. | | S | | 15, 21 | |
| *Anthemis melampodina* L. | | Aqhwan | |  |  | Whole plant | | Antimicrobial. | | S | | 15, 19 | |
| *Anthemis pseudocotula* L. | | Aqhwan | |  |  | Whole plant | | Antimicrobial. | | S | | 15, 19 | |
| *Anvillea gracinii* (Burm.f) DC. | | Nougd | |  |  | Whole plant | | Used treatment for colds, diabetes. | | W, S | | 13 | |
| *Artemisia abyssinica* L. | | Boatheran | |  |  | Whole plant | | Antimicrobial. | | S | | 15, 19 | |
| *Artemisia herbaalba* L. | | Chih | |  |  | Flowery tips | | Digestive disorders, abdominal pain, colic & liver failure. | | S | | 15 | |
| *Artemisia judaica* | | Boaithran | |  |  | Whole plant | | Antipyretic, menstruation regulator, for nerve system, carminative & emmenagogue. | | M | | 12 | |
| *Artemisia monosperma* | | Adar | |  |  | Leaves & flowers | | Constipation, antirheumatic & flu. | | M | | 12 | |
| *Artemisia scoparia* | | Slikah | |  |  | Whole plant | | Purgative, earache, antibacterial, hypercholesteremia, antipyretic, antiseptic, cholagogue, diuretic, vasodilator, jaundice, hepatitis, inflammation of the gall bladder & treatment of scorpion sting. | | M | | 12, 17 | |
| *Artemisia sieberi* Besser*.* | | Chih | |  |  | Leaves | | Used as an anthelmintic. | | S, M | | 12, 15, 27 | |
| *Atractylis carduus* | | Korshoof | |  |  | Whole plant | | Cardiac depression. | | W, S | | 13, 19 | |
| *Calandula micrantha* L. | | - | |  |  | Whole plant | | Central nervous system stimulation. | | S | | 15 | |
| *Carthamus tinctorius* L*.* | | Zafaran | |  |  | Leaves | | Treating red, swollen eye & conjunctivitis. | | S | | 21 | |
| *Centaurea iberica* | | - | |  |  | Leaves | | Treatment of scorpion sting. | | - | | 17 | |
| *Centaurea sinaica* DC. | | Marar | |  |  | Whole plant | | Central nervous system stimulation. | | W, S | | 13, 19 | |
| *Centaurothamnus maximus* | | Byad | |  |  | Leaves | | Wound healing. | | - | | 25 | |
| Table S1: Cont. | | | | | | | | | | | | | |
| **Scientific name** | | **Local name** | | **Family** | | **Part used** | | **Traditional uses** | | **Region** | | **Reference** | |
| *Chrysanthenum coronarium* | | - | | Asteraceae  (Compositae) | | Whole plant | | Blennorrhagia, purgative, inflammation, syphilis, expectorant, stomachic & gonorrhea. | | M | | 12 | |
| *Cichorium intybus* L. | | Hendiban | |  |  | Leaves, seeds & roots | | Antipyretic, dyspepsia, headache, jaundice & as demulcent. | | - | | 21 | |
| *Cnicus benedictus* L*.* | | - | |  |  | Whole plant | | Treatment of scorpion sting. | | - | | 17 | |
| *Conyza bonariensis* L. | | Khoa | |  |  | Whole plant | | Antimicrobial. | | S | | 15, 19 | |
| *Conyza dioscoridis* L. (Desf.) | | Ain alkatkot | |  |  | Leaves | | Epilepsy in children. | | S | | 15 | |
| *Conyza incana* L. | | Arfaj | |  |  | Whole plant | | Central nervous system depression, cardiac stimulation & antimicrobial. | | S | | 15, 19 | |
| *Echinops galalensis* L. | | Karshoof | |  |  | Whole plant | | Antimicrobial. | | S | | 15, 19 | |
| *Echinops hussoni* L. | | - | |  |  | Whole plant | | Antimicrobial. | | S | | 6, 8 | |
| *Echinops spinosissimus* Tuna. | | Shook Al-gamal | |  |  | Whole plant | | Splenic disease & sore throat. | | W, M, S | | 13, 14, 28 | |
| *Eclipta prostrata* L. | | - | |  |  | Whole plant & leaves | | Treatment of scorpion sting. | | - | | 17 | |
| *Euryops arabicus* Steud. | | Kaboor | |  |  | Whole plant | | Cardiac stimulation & for wound healing. | | W, S | | 13, 19, 21 | |
| *Francoeuria crispa* (Forssk) | | Githgath | |  |  | Whole plant | | Used for swellings & as anti-inflammatory. | | M, S | | 14, 28 | |
| *Gnophalium luteo-album* | | - | |  |  | Leaves & stems | | Expectorant, astringent, cholagogue, diuretic, haemostatic & breast cancer. | | M | | 12 | |
| *Jasonia candicans* L. | | - | |  |  | Whole plant | | Cardiac stimulation & antimicrobial. | | S | | 13, 19 | |
| *Kleinia pendula* (Forssk.) DC*.* | | Laban | |  |  | Roots | | Otitis. | | S | | 25 | |
| *Lactuca saligna* | | Odad | |  |  | Whole plant & seeds | | Tonic, carminative, diuretic, colic, catarrh, bronchitis, typhoid, fever & for digestive problem. | | M | | 12 | |
| *Lactuca serriola* L. | | - | |  |  | Latex | | Treatment of scorpion sting. | | - | | 17 | |
| Table S1: Cont. | | | | | | | | | | | | | |
| **Scientific name** | | **Local name** | | **Family** | | **Part used** | | **Traditional uses** | | **Region** | | **Reference** | |
| *Launea nudicaulis* L*.* | | Hwwa | | Asteraceae  (Compositae) | | Leaves | | Antipyretic & antihemorrhagic after childbirth. | | S | | 21 | |
| *Matricaria aurea* (Loel.) Sch.Bip. | | Babunaj | |  |  | Flowers | | Used for making a tea for all stomach ailments & used to treat skin diseases. | | S | | 14, 15, 18 | |
| *Picris abyssinica* L. | | - | |  |  | Whole plant | | Antimicrobial. | | S | | 15, 19 | |
| *Picris cyanocarpa* | | Hozan | |  |  | Whole plant | | Central nervous system stimulation, used for hypertension & cardiac stimulant. | | S | | 15, 19 | |
| *Pluchea arabica* (Boiss.) Qaiser & Lack | | Godot | |  |  | Whole plant | | Treating boils, skin sores & ear infection. | | S | | 15, 18 | |
| *Psiadia arabica* Jaub. | | Tubbak | |  |  | Heated branches | | Antirheumatic & heal broken bones. | | S | | 16 | |
| *Psiadia punctulata* DC. | | Fotaa | |  |  | Branches & stems | | Relieve muscle pain. | | W, S | | 13, 18 | |
| *Pulicaria arabica* L. | | Garaez | |  |  | Whole plant | | For hypertension & antimicrobial. | | S | | 15, 19 | |
| *Pulicaria crispa* | | Arararabi | |  |  | Whole plant | | Antimalarial & stomach disorders. | | S | | 15 | |
| *Pulicaria guestii* Rawi | | - | |  |  | Whole plant | | Strong antimicrobial. | | S | | 15, 19 | |
| *Pulicaria jaubertii* Gamal-Eldin | | Ansif | |  |  | Leaves & flowers | | Used for digestion problem & as tonic. | | S | | 21 | |
| *Pulicaria undulata* (Forssk.) Oliver. | | Gathgath | |  |  | Wole plant | | Central nervous system depression & antimicrobial. | | W, S | | 13, 15, 19 | |
| *Rhanterium epapposum* L. | | Arfaj | |  |  | Whole plant | | Antimicrobial. | | S | | 15, 19 | |
| *Senecio asirensis* Boulos & J. R. I. Wood | | Pedaa, Henna | |  |  | Leaves | | Antipyretic. | | S | | 15, 18 | |
| *Sonchus oleraceus* L. | | Uddaid | |  |  | Leaves & flowers | | Promotes menstruation & treatment of scorpion sting. | | W, M | | 13, 14, 17 | |
| *Tagetes minuta* L. | | Bard-agoosh | |  |  | Leaves & flowers | | Treatment for cold & constipation. | | S | | 16 | |
| *Verbesina encelioides* (Cav.) | | Safeara | |  |  | Leaves | | Wound & skin disease. | | S | | 15 | |
| *Vernonia schimperii* L. | | Neeka | |  |  | Leaves | | Treat scorpion bites, antipyretic & bites. | | S | | 15, 21 | |
|  |  |  |  |  |  | Roots & seeds | | Anthelmintic. | |  |  |  |  |
| Table S1: Cont. | | | | | | | | | | | | | |
| **Scientific name** | | **Local name** | | **Family** | | **Part used** | | **Traditional uses** | | **Region** | | **Reference** | |
| *Xanthium spinosum* L. | | Shobet | | Asteraceae  (Compositae) | | Wole plant | | Antimicrobial. | | S | | 15 | |
| *Alkanna orientalis* (L.) Boiss. | | Lebbed | | Boraginaceae | | Whole plant | | Central nervous system stimulation, antimicrobial & throat pain. | | S | | 19, 21 | |
| *Anchusa milleri L.* | | - | |  |  | Whole plant | | Central nervous system stimulant. | | S | | 19 | |
| *Arnebia hispidissima* (Lehm.) DC. | | Kohaeel | |  |  | Whole plant | | Antipyretic. | | W, S | | 13 | |
| *Cordia myxa* L. | | Bambar | |  |  | Leaves & seeds | | Used for stomach ailments, wounds healer, tonic, expectorant, refreshing, heart and brain diseases & respiratory system. | | M | | 12, 21 | |
| *Cordia sinensis* Lam. | | Tanab | |  |  | Leaves & stems | | Antirheumatic, painful menstruation, bladder diseases, gastric ulcers & malaria. | | - | | 24 | |
| *Echium horridum* L. | | - | |  |  | Whole plant | | For hypertension. | | S | | 19 | |
| *Heliotropium aegyptiacum* Lehm. | | Ramram | |  |  | Root | | Treatment of scorpion sting. | | - | | 17 | |
| *Heliotropium arbainense* Fresen*.* | | Ramram | |  |  | Whole plant | | For hypertension & antimicrobial. | | W, S | | 13, 19 | |
| *Heliotropium digynum* (Forssk) | | - | |  |  | Leaves | | Skin diseases, demonomania & antimicrobial. | | M, W, S | | 12, 13, 19 | |
| *Heliotropium europaeum* L. | | - | |  |  | Whole plant | | Antimicrobial. | | W, S | | 13, 19 | |
| *Heliotropium ramosissimum* DC. | | Rmram | |  |  | Whole plant | | Used for snake bites. | | W | | 13 | |
| *Heliotropium strigosum* Willd. | | Ramram | |  |  | Whole plant | | Used for snake bites. | | - | | 17 | |
| *Trichodesma africanum* L. | | Hamham | |  |  | Whole plant | | Treatment for cough & cold. | | M, S | | 14 | |
| *Balanites aegyptiaca* (L.) Del | | - | | Balanitaceae | | Whole plant | | Treatment of different ailments such as syphilis, jaundice, liver and spleen problems, epilepsy, as treatment for scorpion sting & yellow fever. | | S | | 15, 17 | |
| *Anastatica hierochuntica* L. | | Khaf-Maryam | | Brassicaceae  (Cruciferaceae) | | Whole plant | | Facilitate labor & antidiabetic activity. | | M, S | | 14, 29 | |
| *Brassica rapa* L. | | - | |  |  | Whole plant | | As aphrodisiac. | | S | | 15 | |
| Table S1: Cont. | | | | | | | | | | | | | |
| **Scientific name** | | **Local name** | | **Family** | | **Part used** | | **Traditional uses** | | **Region** | | **Reference** | |
| *Diplotaxis acris (Forssk) Boiss* | | Fegl Algabal | | Brassicaceae  (Cruciferaceae) | | Leaves | | Antidiabetic & wound healing. | | S | | 15 | |
| *Diplotaxis harra L.* | | Harra | |  |  | Whole plant | | Antimicrobial. | | S | | 15 | |
| *Eruca sativa* L. | | Rocka | |  |  | Seeds | | Used in ringworm. | | M, S | | 14 | |
| *Farsetia aegyptiaca* Turra. | | Jarbaa | |  |  | Whole plant | | Antirheumatic. | | W, S | | 13, 14 | |
| *Farsetia longisiliqua* Decne | | - | |  |  | Whole plant | | Central nervous system depression. | | S | | 19 | |
| *Lepidium aucherii* L. | | - | |  |  | Whole plant | | For hypertension & cardiac stimulant. | | S | | 19 | |
| *Lepidium draba* L. | | - | |  |  | Whole plant | | Central nervous system stimulation & antimicrobial. | | S | | 19 | |
| *Lepidium sativum* L. | | Thuffa, Rashad | |  |  | Leaves & seeds | | Used for aches and pains, emollient, antifungal & for measles. | | S | | 30, 21 | |
| *Morettia parviflora* L. | | - | |  |  | Whole plant | | Antimicrobial. | | S | | 15 | |
| *Nasturtium officinale* R.Br. | | Jarjir | |  |  | Whole plant, leaves & roots | | Diuretic, digestive, herpetic eruptions, jaundice & renal disease. | | - | | 20 | |
| *Notoceras bicorne* L. | | - | |  |  | Whole plant | | Antimicrobial. | | S | | 19 | |
| *Savignya parviflora* L. | | - | |  |  | Whole plant | | Central nervous & cardiac systems stimulant. | | S | | 15 | |
| *Schimpera arabica* L. | | - | |  |  | Whole plant | | Cardiac stimulant. | | S | | 19 | |
| *Sisymbrium irio* L. | | - | |  |  | Seeds | | Antipyretic. | | S | | 18 | |
| *Zilla spinosa Prant* L. | | Shibrim | |  |  | Leaves | | Purgative but toxic at high dose. | | S | | 14 | |
| *Commiphora Africana* L. | | - | | Burseraceae | | Whole plant | | Anticancer & anti-inflammatory. | | S | | 21 | |
| *Commiphora habessinica* | | Mrr | |  |  | Resins | | Used for chest pain & as antiinfective. | | - | | 21 | |
| *Commiphora molmol* (Engl.) | | - | |  |  | Barks | | Treatment of snake bites. | | - | | 17 | |
| *Commiphora myrrha* (Nees) Engl*.* | | Myrrha | |  |  | Resins | | Antimicrobial, antiseptic, astringent, carminative, disinfectant, antileishmania & expectorant. | | S | | 15, 27 | |
| *Commiphora opobalsamum* L. | | - | |  |  | Whole plant | | Antimicrobial. | | S | | 19, 15 | |
| *Opuntia ficus-indica* Mill. | | Barshoom | | Cactaceae | | Succulent | | Treatment for pimples & skin problems. | | S | | 15, 16 | |
| Table S1: Cont. | | | | | | | | | | | | | |
| **Scientific name** | | **Local name** | | **Family** | | **Part used** | | **Traditional uses** | | **Region** | | **Reference** | |
| *Tamarindus indica* L. | | Tamr-Hindi | | Caesalpinaceae | | Fruits | | Headache, jaundice, relieve stomach problem, antihypertensive, wound healing & antiemetic. | | S | | 115 | |
| *Celtis Africana* N.L.Burm, | | - | | Cannabaceae | | Leaves & stems | | Antirheumatic, toothache & anticancer. | | - | | 24 | |
| *Cadaba farinose* Forssk. | | Asaf | | Capparaceae  (Capparidaceae) | | Whole herb | | Purgative, anthelmintic, emmenagogue & aperient. | | S | | 8, 24 | |
|  |  |  |  |  |  | Leaves | | A remedy for dysentery, fever, cough & lungs problem. | |  |  |  |  |
| *Cadaba glandulosa* Forssk. | | Qormot | |  |  | Leaves & stems | | As anthelmintic. | | - | | 24 | |
| *Capparis cartilaginea* | | Shafallah | |  |  | Whole plant | | Disinfectant, anti-inflammatory, wound wash, antitumor, tonic & purgative. | | M, S | | 12, 15 | |
| *Capparis decidua* | | Tandhab | |  |  | Whole plant | | Antirheumatic, astringent, cough, tremor, wound, sedative, vermifugal, antidiabetic & gout. | | M, W, S | | 12, 13, 15, 31 | |
| *Capparis spinosa* | | Kabar | |  |  | Roots, barks & leaves | | Aperient, tonic, diuretic, antidiabetic & expectorant. | | M, W, S | | 12, 13, 15, 31 | |
| *Dipterygium glaucum* | | Alqa | |  |  | Leaves | | Expectorant, analeptic & stimulant. | | M, S | | 12, 15 | |
| *Maerua crassifolia* | | Sarh | |  |  | Leaves | | Used in toothache & intestinal disease. | | W, S | | 8, 13 | |
| *Maerua oblongifolia* | | Maru | |  |  | Whole plant | | Hypocholesterolemic. | | S | | 8 | |
| *Dianthus deserti* | | Alhilba | | Caryophyllaceae | | Roots | | Used for sprains. | | W | | 13 | |
| *Polycarpaea repens* | | Rokeka | |  |  | Whole plant | | Used as antidote against snake bites. | | W, S | | 13, 15 | |
| *Catha edulis* Forsk. | | Khat | | Celastraceae | | Leaves | | Central nervous system stimulant & treatment for diabetic. | | S | | 16 | |
| *Moytenus ovatus* | | Hurgran | |  |  | Leaves | | Used for stomach problems. | | W, S | | 13, 20 | |
| *Anabasis articulate* L. | | - | | Chenopodiaceae | | Whole plant | | Cardiac stimulation & antihypertension. | | S | | 19 | |
| Table S1: Cont. | | | | | | | | | | | | | |
| **Scientific name** | | **Local name** | | **Family** | | **Part used** | | **Traditional uses** | | **Region** | | **Reference** | |
| *Anabasis setifera* Moq. | | Himd | | Chenopodiaceae | | Leaves | | Antidepressant. | | M, S | | 14, 15 | |
| *Atriplex halimus* L. | | Rughl | |  |  | Seeds | | Emetic. | | W | | 13, 20 | |
| *Beta vulgaris* | | - | |  |  | Roots | | Headache, toothache, liver pain, burns, constipation, emmenagogue, purgative, eye inflammation, itch, scurf & dandruff, tumor, leukemia, anemia, snake bite, vermifugal & antirheumatic. | | M | | 12 | |
| *Brassia eriophora* | | - | |  |  | Whole plant & seeds oil | | Antirheumatic, snake bite & vermifugal. | | M | | 12 | |
| *Brassia muricata* | | - | |  |  | Whole plant & seeds oil | | Kidney diseases, antirheumatic & ulcer gargle. | | M | | 12 | |
| *Chenopodium album* L. | | Atrah | |  |  | Leaves & fruits | | Postnatal problems. | | W, M, S | | 13, 14, 15 | |
| *Chenopodium ambrosioides* L. | | - | |  |  | Leaves | | Used as treatment for scorpion sting. | | - | | 17 | |
| *Chenopodium murale* L*.* | | AlZorbiah, Jkheara | |  |  | Whole plant & Flowers | | Stomachache, leishmaniasis, antitumor & antihypertension. | | M, W, S | | 12, 13, 15, 32 | |
| *Cornulaca monacantha* Delile | | Had | |  |  | Leaves | | Used for liver problems, jaundice & purgative. | | W, S | | 13, 15 | |
| *Haloxylon salicornicum* | | Ramath | |  |  | Whole plant | | Antidiabetic & used for cold. | | W, M, S | | 13, 14, 15 | |
| *Kochia indica* | | - | |  |  | Whole plant | | Heart tonic. | | M | | 12 | |
| *Salsola imbricate* | | Kha^,^reet | |  |  | Whole plant | | Anthelmintic. | | M, S | | 14, 15 | |
| *Seidletzia rosmarinus* Bunge ex Boiss | | Ushnan. | |  |  | Leaves | | Antimicrobial. | | S | | 19 | |
| *Suaeda vera* L. | | - | |  |  | Whole plant | | Cardiac stimulant. | | S | | 15 | |
| *Ceratophyllum demersum* L. | | - | | Ceratophyllaceae | | Whole plant | | Treatment for scorpion sting. | | - | | 17 | |
| *Cleome amblyocarpa* | | Khunayzah | | Cleomaceae | | Whole plant | | Antimicrobial. | | W, S | | 13, 15 | |
| *Cleome arabica* | | Zafrah-Amal | |  |  | Leaves | | Tonic, stimulant, appetizer, purgative & for panicula. | | M, S | | 12, 15 | |
| *Cleome brachycarpa* | | Birbran | |  |  | Whole plant | | Itching, antirheumatic, inflammation, leukoderma & skin disease. | | M, S | | 12, 15 | |
| *Cleome chrysantha* | | Safaira^,^a | |  |  | Whole plant | | Anthelmintic & antiseptic. | | S | | 8 | |
| Table S1: Cont. | | | | | | | | | | | | | |
| **Scientific name** | | **Local name** | | **Family** | | **Part used** | | **Traditional uses** | | **Region** | | **Reference** | |
| *Cleome gynandra* L. | | - | | Cleomaceae | | Whole plant | | Treatment for scorpion sting. | | - | | 17 | |
| *Cleome viscosa* L. | | Om-Hanif | |  |  | Whole plant | | Carminative, anthelmintic & rubefacient. | | S | | 8 | |
| *Hypericum chrysostrictum* L. | | - | | Clusiaceae | | Whole plant | | Central nervous system depression & antimicrobial. | | S | | 19 | |
| *Commelina benghalensis* L*.* | | - | | Commelinaceae | | Whole plant | | Treatment for scorpion sting. | | - | | 17 | |
| *Convolvulus arvensis* L. | | Olaique | | Convolvoulaceae | | Leaves & fruits | | Foot cracking. | | W, M, S | | 13, 14, 15 | |
| *Convolvulus fatmensis* | | Al-oleeq | |  |  | Leaves | | Anti-inflammatory. | | S | | 15 | |
| *Convolvulus hystrix* | | - | |  |  | Whole plant | | Antimicrobial. | | S | | 15 | |
| *Convolvulus oxyphyllus* | | - | |  |  | Whole plant | | Cardiac depression. | | S | | 15 | |
| *Convolvulus pilosellifolius* | | - | |  |  | Whole plant | | Antimicrobial. | | S | | 15 | |
| *Cressa cretica* | | Naduoh | |  |  | Whole plant | | Digestive, tonic, vermifugal, antiasthmatic, tuberculous, hematonic, CNS depressant & appetizer. | | M, S | | 12, 19 | |
| *Evolvulus alsinoides* L. | | - | |  |  | Whole plant | | Treatment of scorpion sting. | | - | | 17 | |
| *Ipomoea aquatic* Forssk. | | - | |  |  | Leaves | | Treatment of scorpion sting. | | - | | 17 | |
| *Ipomoea eriocarpa* R.Br. | | - | |  |  | Leaves | | Treatment of scorpion sting. | | - | | 17 | |
| *Citrullus colocynthis* L. | | Hunzal | | Cucurbitaceae | | Roots, seeds & fruits | | Used as diuretic, emetic, expectorant, purgative, jaundice, ascites, analgesic, anesthetic, anti-HIV, antiaging, allergy, antiasthmatic, antibacterial, antidiabetic, sedative, antihemolyteic, antimalaria, antimitotic, antioxidant, antiscorbutic, antiseptic, antitumor, carminative, fungicide, herbicide, insecticide, laxative & lubricant. | | M, W, S | | 12, 15 | |
| *Coccinia grandis* L. | | Bakhra^,^a | |  |  | Leaves | | Treat earache & for scorpion sting. | | S | | 16, 17 | |
| Table S1: Cont. | | | | | | | | | | | | | |
| **Scientific name** | | **Local name** | | **Family** | | **Part used** | | **Traditional uses** | | **Region** | | **Reference** | |
| *Cucumis melo* | | Shamam | | Cucurbitaceae | | Leaves & fruits | | Astringent, demulcent, laxative, antipyretic, jaundice & renal calculi. | | - | | 20 | |
| *Cucumis prophetarum* | | Shree- Elzeeb | |  |  | Roots, seeds & fruits | | Sexual diseases, liver diseases, stomachache, demonomania, emetic, antimicrobial & purgative. | | M, S | | 12, 15 | |
| *Kedrostis foetidissima* Jacq. Cong*.* | | - | |  |  | Leaves | | Used for warts. | | S | | 25 | |
| *Momordica balsamina* L. | | Madoda | |  |  | Whole plant | | Antimicrobial. | | S | | 19 | |
| *Juniperus polycarpos* L. | | - | | Cupressaceae | | Whole plant | | CNS stimulant & antimicrobial. | | S | | 15 | |
| *Juniperus procera* | | Arar | |  |  | Leaves | | Gout, jaundice & cure wounds. | | S, W | | 33 | |
|  |  |  |  |  |  | Fruits | | The smoke of fruiting branches used as antirheumatic, headaches & skin diseases. | |  |  |  |  |
|  |  |  |  |  |  | Twigs & buds & small branches | | Intestinal worms and a decoction of dry young branches is used as medicine against itch of camels. | |  |  |  |  |
|  |  |  |  |  |  | Resin | | Stimulant and for treatment of ulcers and liver diseases. | |  |  |  |  |
| *Cuscuta campestris* | | - | | Cuscutaceae | | Whole plant | | Purgative & constipation. | | M | | 12 | |
| *Cynomorium coccineum* L. | | Tartooth | | Cynomoriaceae | | Whole plant | | Astringent, aphrodisiac, laxative & tonic. | | M | | 12, 15 | |
| *Cyperus longus* L*.* | | - | | Cyperaceae | | Whole plant | | Treatment of scorpion sting. | | - | | 17 | |
| *Cyperus rotundus* L. | | Alsaad | |  |  | Whole plant | | Treatment of scorpion sting. | | W | | 13, 17 | |
| *Ephedra alata* L. (female) | | - | | Ephedraceae | | Whole plant | | Central nervous system stimulant & antimicrobial. | | S | | 19 | |
| *Acalypha ciliata* Forssk | | - | | Euphorbiaceae | | Leaves | | Antimalaria, anti-scabies & anthelmintic. | | S | | 25 | |
| *Acalypha fruticosa* Forssk | | Zohar | |  |  | Leaves | | Used for treating bee stings. | | S | | 15 | |
| *Acalypha indica* L. | | Thfelan | |  |  | Whole plant | | Used for cure from bronchitis, pneumonia & asthma. | | S | | 15 | |

| Table S1: Cont. | | | | | | |
| --- | --- | --- | --- | --- | --- | --- |
| **Scientific name** | **Local name** | **Family** | **Part used** | **Traditional uses** | **Region** | **Reference** |
| *Andrachne aspera* Spreng. var. glandulosa A.Rich | Kamas | Euphorbiaceae | Whole plant | Used in eye problems & for eye wash. | S | 9 |
| *Chrozophora oblique* L. | - |  | Whole plant | Antimicrobial. | S | 15 |
| *Chrozophora oblongifolia* (DC.) A. Juss. ex Spreng | Tannoum |  | Whole plant | Antimicrobial, cathartic & emetic. | S | 15 |
| *Chrozophora plicata* (Vahl) A. Juss | Tanoom |  | Whole plant | Depurative & purgative. | S | 8, 18 |
|  |  |  | Leaves | Used for the cure of leprosy. |  |  |
| *Clutia lanceolata* Forssk*.* | Laukh |  | Whole plant | Hypoglycemic. | S | 9 |
| *Croton lobatus* L. | - |  | Leaves | Used as treatment of scorpion sting. | - | 17 |
| *Euphorbia arabica* Hochst. | - |  | Whole plant | Used in skin infection. | S | 34 |
| *Euphorbia cuneate* Vahl. | AL-baky |  | Whole plant | Sedative & antimicrobial. | W, S | 13, 9 |
| *Euphorbia cyparissioides* L. | - |  | Whole plant | Antimicrobial. | S | 19 |
| *Euphorbia dracunculoides* Lam. | Yaktin |  | Leaves & fruits | Purgative, intestinal disorders, antitumor & wart remover. | M, S | 12, 8 |
| *Euphorbia granulate* Forssk. | Lebbein |  | Whole plant | Blood purifier, diuretic, purgative & vermifugal. | M, W, S | 12, 13, 53 |
|  |  |  | Latex | Used as purgative. |  |  |
| *Euphorbia helioscopia* L. | Emaiah |  | Whole plant | Purgative, ulcer, antirheumatic, vermifugal, cholera, cancer, anthelmintic, catarrh & eruptions, neuralgia. | M, S | 8, 12 |
| *Euphorbia hirta* L. | - |  | Whole plant | Antiasthmatic, antiasthmatic & bronchitis. | S | 15 |
| *Euphorbia peplus* L. | Khaneez |  | Whole plant | Antihypertension. | W, S | 13, 15 |

| Table S1: Cont. | | | | | | |
| --- | --- | --- | --- | --- | --- | --- |
| **Scientific name** | **Local name** | **Family** | **Part used** | **Traditional uses** | **Region** | **Reference** |
| *Euphorbia retusa* Forssk. | Ghazalah | Euphorbiaceae | Whole plant | Antitussive, antiasthmatic & mentalia disorders. | M, S | 12, 14, 15 |
|  |  |  | Latex | Eczema, wound healing & for leishmaniasis. |  |  |
| *Euphorbia schimperiana* Scheele*.* | Saibarisodis |  | Whole plant | Antitussive, antiasthmatic, earache, skin infections & snake bites. | S | 35 |
| *Euphorbia scordifolia* Jacq. | Rummid |  | Whole plant | Antipyretic & constipation. | S | 34 |
| *Euphorbia terracina* L. | Terasina-harmal |  | Whole plant | Used as a remedy for fever & paralysis. | S | 34 |
| *Jatropha curcas L.* | Kharat |  | Leaves | Used in wounds, eczema & scabies. | S | 9 |
| *Jatropha glauca* | Obeeb |  | Whole plant | Treatment of chronic skin diseases. | S | 9 |
| *Jatropha pelargoniifolia* | Obab |  | Whole plant & petioles | The sap of the petiole is applied to ulcers. | S | 15 |
| *Phyllanthus maderaspatensis* L*.* | Tamarhindi |  | Leaves | Used for headache. | S | 8 |
| *Ricinus communis* L*.* | Kharwah |  | Whole plant | Treatment of scrofulous sores, boils & rheumatic swellings | W, S | 13 |
| *Acacia arabica* L. | - | Fabaceae  (Leguminosae) | Bark, gum, leaves, seeds & fruits | Nutritive, expectorant, antihemorrhagic, diarrhea, cough, blennorrhagia, mouthwash, astringent, dysentery, gargle, tonic, inflammation of urinary tract, hemorrhagic, transudation, aphrodisiac & diabetes. | S, M | 18, 12 |
| *Acacia ehrenbergianan* | Salam |  | Wood | Used to treating paralysis. | - | 20 |
| *Acacia farnesiana* | - |  | Whole plant | Vermifugal, dysentery, mouth wash, blood diseases, antipruritic, ulcers, leukoderma, catarrh, vulvovaginitis, astringent, demulcent, aphrodisiac, antispasmodic & insecticidal. | M | 12 |
| Table S1: Cont. | | | | | | |
| **Scientific name** | **Local name** | **Family** | **Part used** | **Traditional uses** | **Region** | **Reference** |
| *Acacia gerardi* Benth. | Karat | Fabaceae  (Leguminosae) | Resin & pods | Used for burns, toothache & antipyretic. | - | 20 |
| *Acacia seyal* | Talh |  | Barks, gums & seeds | Astringent, cold, ophthalmia, diarrhea, haemorrhage & leprosy. | M | 12 |
| *Acacia negrii* Pichi. | Salam |  | Leaves | Treat eye diseases. | S | 16 |
| *Acacia oerfota* Forssk. | - |  | Whole plant | Treatment of scorpion sting. | - | 17 |
| *Abrus precatorius* L. | Habb shoush |  | Root | Used to treatment of scorpion sting, emetic, eye disease & purgative. | - | 17, 20 |
| *Albizia lebbeck* | - |  | Fruits & stems | Used for snake bites, mouthwash, antiparalysis, night blindness, astringent, diarrhea, dysentery, gonorrhea & swelling of cervical glands. | M | 12 |
| *Alhagi graecorum* Boiss | Aqool |  | Whole plant | Analgesic, antitussive, antihemorrhoids, anti- rheumatic, aphrodisiac, diuretic & laxative. | S | 15 |
| *Alhagi maurorum* Medic. | Al-Agool |  | Leaves | Antioxidant & analgesic. | S | 15 |
| *Alhagi melorum* | - |  | Whole plant | Antipyretic, digestive, tonic, purgative, diuretic & catarrh. | M | 12 |
| *Astragalus atropilosus* | - |  | Leaves | For backache. | S | 15 |
| *Astragalus mareoticus* | - |  | Leaves | Treatment of scorpion sting. | - | 17 |
| *Astragalus sieberi* | - |  | Whole plant | Antihypertension. | S | 19 |
| *Astragalus spinosus* (Forssk.) Muschl. | Katad |  | Whole plant | Treat leukemia & promote wound healing. | W, S | 13, 15 |
| *Astragalus tribuloides* | - |  | Seeds | Anti-esophagitis, anti-enteritis & peri colitis. | M | 12 |
| *Cassia holosericea* | - |  | Leaves & fruits | Tonic for digestive system, flatulence & purgative. | M | 12 |
| *Cassia senaa* L. | Senna |  | Whole plant | Antimicrobial, laxative & purgative. | S | 19, 16 |

| Table S1: Cont. | | | | | | |
| --- | --- | --- | --- | --- | --- | --- |
| **Scientific name** | **Local name** | **Family** | **Part used** | **Traditional uses** | **Region** | **Reference** |
| *Cassia italic* Mill. | Ishriq | Fabaceae  (Leguminosae) | Leaves | Laxative, treating influenza and other respiratory disease & urinary tract purifier. | M, S | 15, 16 |
| *Cicer arietinum* L. | Himas |  | Seeds | Used in abortion, cure ulcer, antitumor, anti-scabies, for pimples, toothache, oedemas, renal calculi, aphrodisiac & diuretic. | - | 20 |
| *Clitoria ternatea* L. | - |  | Leaves, roots & stems | Treatment of scorpion sting. | - | 17 |
| *Crotalaria retusa* L. | - |  | Stem | Treatment of scorpion sting. | - | 17 |
| *Delonix elata* L. | Ranf |  | Leaves & seeds. | Mosquito control agent. | S | 15 |
| *Desmodium gangeticum* | - |  | Roots | Treatment of scorpion sting. | - | 17 |
| *Dichrostachys cinereal* L. | - |  | Roots & leaves | Treatment of scorpion sting. | - | 17 |
| *Glycyrrhiza glabra* L. | Irk al hiel |  | Rhizomes | Treating muscle pain & treatment of scorpion sting. | S | 17, 18 |
| *Indigofera articulate* Gouan. | Khedaish |  | Roots | Relieve toothache. | M, S | 12, 18 |
|  |  |  | Whole plant | Purgative, diuretic, lithotomy, antitoxicant, swelling of spleen, antitumor, antirheumatic, teeth protection & snake bite. |  |  |
| *Indigofera oblongifolia* | Hasar |  | Roots & Leaves | Analgesic, remove hair dandruff & anti-inflammatory. | S | 20 |
| *Indigofera tinctorial* L. | - |  | Whole plant | Treatment of scorpion sting. | - | 17 |
| *Lablab purpureus* L. | Lablab |  | Roots | Laxative, diuretic & regulate menstruation. | S | 20 |

| Table S1: Cont. | | | | | | |
| --- | --- | --- | --- | --- | --- | --- |
| **Scientific name** | **Local name** | **Family** | **Part used** | **Traditional uses** | **Region** | **Reference** |
| *Medicago sativa* L. | Jat | Fabaceae  (Leguminosae) | Leaves | Used for bone fracture, bruises reliever & used as an aphrodisiac. | S | 20 |
| *Melilotus albus* Medik. | Otrah |  | Whole plant | Astringent & as antirheumatic. | W, S | 20 |
| *Melilotus indicus* L. | Handaquq |  | Whole plant | Emollient. | - | 20 |
| *Melilotus officinalis* | Iklilulmalik |  | Flowers, stems & roots | Diuretic, scabies, boils, wounds, for edema, insomnia & colic. | S | 20 |
| *Ononis serrate* L. | - |  | Whole plant | Antimicrobial. | S | 19 |
| *Prosopis cineraria* L. | Ghaf |  | Whole plant | Treatment of scorpion sting. | - | 17 |
| *Prosopis juliflora* L. | - |  | Whole plant | Antiprotozoal. | S | 18 |
| *Psoralea plicata* L. | - |  | Whole plant | Antimicrobial. | S | 19 |
| *Raetam ratam* L. | - |  | Whole plant | Anticancer & anti-inflammatory. | S | 27 |
| *Senna alexandrina* Mill. | Sana |  | Leaves & fruits | Stimulant laxative & cathartic. | S | 15 |
| *Senna italic* Mill. | Sana-mekki |  | Leaves & fruits | For elephantiasis & ophthalmic diseases. | M, W, S | 12, 13, 15 |
| *Taverniera lappacea* L. | - |  | Whole plant | Central nervous system stimulation & antimicrobial. | S | 15, 19 |
| *Tephrosia apollinea* Del. | Dhafran |  | Whole plant | Antihypertension & cardiac stimulant. | S | 15, 19 |
| *Tephrosia nubica ssp. Arabica* (Boiss.) Gillet. | - |  | Whole plant | Antihypertension & cardiac stimulant. | S | 15 |
| *Tephrosia purpurea* | Sakhal |  | Seeds | Relieve urinary tract problems. | S | 16 |
| *Trigonella anguina* L*.* | Nafel |  | Whole plant | Antimicrobial. | S | 19 |
| *Trigonella foenum-graecum* L. | Helba |  | Seeds | Relieve upset stomach. | S | 16 |
| *Trigonella stellate* Forssk. | Girgas |  | Whole plant | Hair diseases. | N | 14 |
| *Frankenia pulverulenta* | - | Frankenaceae | Whole plant | Carminative & analgesic. | M | 12 |

| Table S1: Cont. | | | | | | |
| --- | --- | --- | --- | --- | --- | --- |
| **Scientific name** | **Local name** | **Family** | **Part used** | **Traditional uses** | **Region** | **Reference** |
| *Fumaria parviflora* | Humaida | Fumariaceae | Whole plant | Digestive, increase biliary secretion, antipyretic, antitoxic, hematonic, diuretic, appetizer, blood purifier, skin diseases, spleen disorder, purgative & antiemetic. | M | 12 |
| *Geranium trilophum* Boiss. | Zahra^,^a | Geraniaceae | Whole plant | Relieve backache. | S | 16 |
| *Avena sativa* L. | Shofan | Gramineae  (Poaceae) | Colloidal oat extract. | Skin diseases. | S | 8 |
| *Chloris virgata* | - |  | Whole plant | Tonic. | M | 12 |
| *Cutandia memphitica* L. | - |  | Whole plant | Central nervous system stimulant & antimicrobial. | S | 15 |
| *Cymbopogon schoenanthus* L. | El-lemad |  | Whole plant | Antipyretic, colic, antispasmodic, hypotension, carminative, colic gripes, flatulence, polyarthritis, hysteritis, analgesic, sedative & expectorant. | M, S | 12, 15 |
| *Cynodon dactylon* | Thail |  | Whole plant, roots & juice | Diuretic, astringent, ophthalmic disorders, hemorrhage, rhinorrhagia, dysentery, urinary tract inflammation & dysuria. | M, W, S | 12, 13, 14, 15 |
| *Dactylotenium aegyptium* | Bahma |  | Whole plant & seeds | Analgesic & wound sepsis. | M, S | 12, 14, 19 |
| *Echinochloa colona* | - |  | Whole plant | Digestive, constipation, treatment of scorpion sting & increase bile secretion. | M, W | 12, 17 |
| *Eleusine indica* | - |  | Whole plant | Antispasmodic, antipyretic &liver diseases. | M | 12 |
| *Heteropogon contortus* L. | - |  | Whole plant | Treatment of scorpion sting. | - | 17 |
| Table S1: Cont. | | | | | | |
| **Scientific name** | **Local name** | **Family** | **Part used** | **Traditional uses** | **Region** | **Reference** |
| *Imperata cylindrica* | Halfa | Gramineae  (Poaceae) | Roots, flowers, branches, young buds | Antipyretic, diuretic, hemorrhagic, hemolysis, rhinorrhagia, carminative, astringent, tonic, emollient, hematuria, hematemesis, edema, jaundice, antibacterial, cancer & tonic. | M, W | 12, 13 |
| *Panicum turgidum* Forssk. | Tammam |  | Whole plant | Eye infection. | W, M, S | 13, 14, 15 |
| *Phragmites australis* Cav. | Hajna |  | Whole plant | Used as antiemetic & antipyretic. | - | 20 |
| *Setaria viridis* (L.) P. Beauv. | - |  | Leaves | Treatment of scorpion sting. | - | 17 |
| *Ribes nigrum* L. | - | Grossulariaceae | Fruits | For throat inflammation & respiratory tract ailment. | - | 24 |
| *Iris germanica* L. | - | Iridaceae | Roots | For treatment of cancer, inflammation, bacterial & viral infections. | - | 24 |
| *Lanata camara* L. | - | Labiatae  (Lamiaceae) | Whole plant | Antipyretic, antimicrobial & antimutagenic. | S | 15 |
| *Lavandula coronopifolia* | Dikta |  | Whole plant | Antibacterial. | S | 9 |
| *Lavandula dentata* L. | Dhurum |  | Leaves & flowers | Used in headache, relieve rheumatic pain & cold. | W, S | 13, 16 |
| *Lavandula pubescens* Decne. | Attan |  | Leaves & flowers | For cold & headache. | W, S | 16 |
| *Lavandula stoechas subsp. Stoechas* | Lavender |  | Whole plant | antiseptic, antispasmodic, digestive, expectorant & antiasthmatic. | S | 9 |
| *Marrubium vulgare* L. | Frasyoon |  | Whole plant | Used for the treatment of coughs, and chronic bronchitis, dyspepsia, jaundice amenorrhea, rheumatism & hepatitis. | W, S | 13, 19 |
| *Mentha lavendulaceae* Willd. | Niena^,^a |  | Whole plant | Analeptic & carminative. | S | 15 |
| *Mentha longifolia* L. | Haback |  | Leaves | Used for headache, antipyretic, menstrual cramps & anti-infective. | W, S | 13, 16 |
| Table S1: Cont. | | | | | | |
| **Scientific name** | **Local name** | **Family** | **Part used** | **Traditional uses** | **Region** | **Reference** |
| *Mentha microphylla* C. Koch | Niena^,^a, bariniena^,^a | Labiatae  (Lamiaceae) | Whole plant | Analeptic, appetizer & carminative. | W, S | 13, 34 |
| *Meriandra benghalensis* Benth. | Dharah |  | Leaves | Used for headache, joint ache & muscle pain & skin problems. | S | 16 |
| *Nepeta deflersiana* | Shya^,^a |  | Leaves | Relieve stomach ache, antimicrobial & in burns. | S | 16, 19 |
| *Ocimum americanum* L. | Sims |  | Leaves | Used in parasitic skin disease. | S | 15 |
| *Ocimum basilicum* L. | Rayhan |  | Leaves | Allay upset stomach ache, cold & in fever. | W, S | 13, 16 |
| *Ocimum tenuiflorum* L. | Shajrat-azzir |  | Whole plant | Snake bites & scorpion sting. | S | 15 |
|  |  |  | Leaves | Used in cough & bronchitis. |  |  |
| *Origanum majorana L.* | Bardakush |  | Whole plant | Used in asthma, cough, indigestion, rheumatism & headache. | S | 9 |
| *Origanum syriacum L.* | Al-barda |  | Leaves | Antitussive & anti-inflammatory. | S | 15 |
| *Oriranum syriacum var. bevanii L* | Oregano |  | Whole plant | Treating tooth decay, gum infections & cough. | S | 15 |
| *Otostegia fruticose* Forssk. | Shakab |  | Leaves & flowers | Irritation of eye, as remedy for sun stroke & for gout. | W, S | 15, 16 |
| *Plectranthus asirensis* | Shar Elkrood |  | Leaves | Used to treat diaper rash & itching. | S | 16 |
| *Plectranthus barbatus* Andres. | Shar Elkarood |  | Leaves | As deodorant. | S | 15 |
| *Plectranthus cylindraceus* | Khurub |  | Whole plant | A remedy for sore throat. | S | 15 |
| *Plectranthus tenuiflorus* | Shar |  | Leaves | Used for earache. | S | 16 |
| *Rosmarinus officinalis* L. | Eklel Aljabal |  | Whole plant | Antifungal. | S | 15 |
| *Salvia aegyptiaca* | Ghashba |  | Whole plant | Eye diseases, diarrahoea & blennorrhagia. | W, M, S | 8, 12 |
| Table S1: Cont. | | | | | | |
| **Scientific name** | **Local name** | **Family** | **Part used** | **Traditional uses** | **Region** | **Reference** |
| *Salvia lanigera* L. | Jurayba | Labiatae  (Lamiaceae) | Whole plant | Carminative & used in indigestion. | S | 34 |
| *Salvia spinose* L. | Harsha |  | Seeds | Used for the cure of toothache, gonorrhea & urethritis. | S | 8 |
| *Stachys Sp. Aff. Schimperi* Vatke | - |  | Whole plant | Strong antimicrobial activity. | S | 19 |
| *Teucrium oliverianum* Ging. | Qassapa |  | Whole plant | Diabetes. | M, S | 14, 15 |
| *Teucrium polium* L. | Jaada |  | Whole plant | Treat liver disease, jaundice, diabetes, fertility problems & cancer. | W, S | 36 |
| *Teucrium yemense* Defl. | Rechal Fatimah |  | Whole plant | Used as anti-diabetic & in kidney problems. | S | 15 |
| *Thymbra spicata subsp. Spicata* L. | Za^,^atar |  | Whole plant | Antimicrobial. | S | 15 |
| *Thymus decussates Benth.* | Za^,^atar |  | Whole plant | Antiemetic. | S | 15 |
| *Thymus vulgaris* L. | Za^,^atar |  | Whole plant | Antiseptic, anthelmintic, expectorant, carminative, diuretic, sedative, used in veterinary medicine. | S | 15 |
| *Allium ampeloprasum* | - | Liliaceae | Leaves | Antimicrobial. | S | 15 |
| *Allium cepa* | Basl |  | Bulb | Diabetes, colic gripes, flu, catarrh, bronchitis, ulcers, dysentery, antiepileptic, rhinorrhagia, jaundice, diuretic, ophthalmia & demonomania. | M | 12 |
| *Allium sativum* | Thom |  | Bulb | Antiasthmtic, increase blood circulation, muscle relaxation, diabetes, catarrh, bronchitis, flu, dysentery, hypertensive, urinary tract inflammation, liver diseases, antirheumatic, diuretic, emmenagogue, diarrhea & antiemetic. | M | 12 |
| Table S1: Cont. | | | | | | |
| **Scientific name** | **Local name** | **Family** | **Part used** | **Traditional uses** | **Region** | **Reference** |
| *Asparagus africanus* Lam. | Khurus | Liliaceae | Leaves | Relieve breathing problems. | W, S | 16, 15 |
| *Cinnamon zellanicum* L. | Qerphah | Lauraceae | Whole plant | Antimicrobial. | S | 15 |
| *Linum usitatissimum* L. | Hab kattan | Linaceae | Seeds | Constipation, painful joint, urinary disorder & venereal diseases. | - | 20 |
| *Lawsonia inermis* L. | Henna | Lythraceae | Branches, leaves, flowers & young buds | Gargle, spleen tumor, skin diseases, hair tonic, headache & jaundice. | M, S | 12, 16 |
| *Syzygium aromaticum* L*.* | Mesmar |  | Whole plant | Treat toothache, respiratory disorders, inflammation & gastrointestinal disorders. | S | 18 |
| *Abutilon pannosum* G. Forst. | - | Malvaceae | Whole plant | Antimicrobial. | S | 19 |
| *Gossypium barbadense* L. | Khutin |  | Seeds | Treatment for earache. | S | 16 |
| *Hibiscus europaeum* L. | Raein |  | Whole plant | Antimicrobial | S | 15 |
| *Hibiscus sabdariffa* L. | Karkeda |  | Whole plant | Antihypertension, reduce the testicular damage & ameliorate the drop-in sperm quality. | S | 15 |
| *Malva parviflora* L. | Khobaiza |  | Whole plant | Laxative & promotes hair growth. | M, S, W | 13, 14, 15 |
| *Azadirachta indica* A. | - | Meliaceae | Whole plant | Antifungal. | W, S | 15, 30 |
| *Cocculus hirsutus* L. | Hamr almajun | Menispermaceae | Leaves & roots | Used as febrifuge, emetic, demulcent, for digestive problem & purgative. | S | 20 |
| *Cocculus pendulus* | - |  | Whole plant | Antipyretic. | M | 12 |
| *Albizzia lebbeck* L. | Lebbeck | Mimosaceae | Phloem | Anthelmintic. | M | 12 |
| *Dorstenia foetida* Forsskal. | Kartib | Moraceae | Seeds | Stomach disorders. | - | 20 |
| *Ficus carica* L. | Teen |  | Leaves, fruits & latex | Used for burns, leprosy, tonic & as diuretic. | W | 20 |
| *Ficus palmata* Forssk. | Hamat |  | Latex | Yogurt production. | W, S | 12, 16 |
| *Ficus salicifolia* | Lithab |  | Whole plant | Leukoderma & eye wash. | M, S | 12, 19 |
| Table S1: Cont. | | | | | | |
| **Scientific name** | **Local name** | **Family** | **Part used** | **Traditional uses** | **Region** | **Reference** |
| *Moringa peregrine* Forssk. | Habb Elyasar | Moringaceae | Seeds | Analgesic, abdominal pain, burns, constipation, laxative & for headache. | S | 37 |
| *Eucalyptus camaldulensis* Dehnh. | Khafour | Myrtaceae | Whole plant | Abortion & perfume. | M, S | 14, 15 |
| *Eucalyptus dives* L. | Eucalyptus |  | Whole plant | Strong antiseptic & antiviral activity. | S | 15 |
| *Eucalyptus globules* L. | - |  | Whole plant | Antifungal. | S | 15 |
| *Myrtus communis* L. | Hadas |  | Leaves | Used for abdominal colic, antipyretic, cough & as insecticidal. | - | 20 |
| *Pimenta dioica* L. | - |  | Whole plant | Anti-inflammatory, analgesic & antipyretic. | S | 15, 38 |
| *Boerhavia coccinea* | - | Nyctaginaceae | Roots | Diuretic & urinary tract disorders. | M | 12 |
| *Commicarpus grandifloras* | - |  | Whole plant | Strong antimicrobial activity. | S | 19 |
| *Jasminum grandiflorum* L. | Anbar, Yasmin | Oleaceae | Leaves & flowers | Treatment for dysentery, abdominal pain & colic. | S | 20 |
| *Olea european* L. | Athm |  | Twigs & branches | Used as tooth brush & keeping gums healthy, liver diseases, ulcer, for edema & diabetes. | W, S | 13, 16, 20 |
| *Epilobium hirsutum* L. | Saqalqurab | Onagraceae | Whole plant | Central nervous system depression & antimicrobial. | S | 19 |
| *Cistanche tubulosa* | Dhanun | Orobanchaceae | Leaves, stems & flowers | Used for jaundice & diarrahoea. | S, M | 12, 21 |
| *Lindenbergia sinaica* L. | - |  | Whole plant | Antimicrobial. | S | 19 |
| *Eulophia petersii* Reichb.f. | Iseb | Orchidaceae | Basal stem bulbs | Used to treat skin problems. | S | 20 |
| *Oxalis corniculate* L. | - | Oxalidaceae | Leaves & flowers | Antiparasitic, antivertigo & mouth inflammation. | S | 25 |
| *Argemone Mexicana* L. | Argemonia | Papavaraceae | Whole plant | Antimicrobial. | W, S | 19 |
| *Agemone ochroleuca* Sweet | - |  | Whole plant | Strong antimicrobial activity. | W, S | 19 |
| *Papaver somniferum* Schl. | Hashish |  | Capsules & seeds | Antitussive & insomnia. | S | 15, 20 |

| Table S1: Cont. | | | | | | |
| --- | --- | --- | --- | --- | --- | --- |
| **Scientific name** | **Local name** | **Family** | **Part used** | **Traditional uses** | **Region** | **Reference** |
| *Sesamum indicum* L. | Gilgilan | Pedaliaceae | Seed oils | Used as treatment for dysentery, colic & urinary problems. | S | 20 |
| *Plantago ammplexiculis* Cav. | Rabal | Plantaginaceae | Whole plant & leaves | Renal disease & urinary tract purifier. | M, S | 14 |
| *Plantago coronopus* L. | Rebla |  | Whole plant | Used as laxative & wound healing. | - | 20 |
| *Plantago major* L. | Lisan Alkalb |  | Leaves & seeds | Used for diarrhea, dysentery, ulcer & abscesses. | - | 20 |
| *Plantago ovata* Forssk. | Geneima |  | Seeds | Used as a laxative, an emollient, demulcent and astringent & particularly in chronic colitis. | S | 15 |
| *Limonium axillare* Forssk. | Kattaf | Plumbaginaceae | Whole plant | Central nervous system depression & antimicrobial. | S | 15 |
| *Plumbago zeylanica* L. | Ensain |  | Whole plant | Antirhumatic, dysmenorrhea, carbuncles, contusion of the extremities, ulcers & elimination of intestinal parasites. | S | 15 |
| *Calligonum comosum* L. | Arta^,^a | Polygonaceae | Whole plant | Anti-inflammatory & antiulcer activity. | S | 15 |
| *Emex spinous* L. | Hambazz |  | Whole plant | Purgative, diuretic, digestive, appetizer, stomach troubles & anthelmintic. | M, S | 12, 15, 18 |
| *Polygonum argyrocoleum* Steud. | Abuzalaf |  | Whole plant | Used in stomach troubles. | S | 34 |
| *Rheum palmatum* L. | - |  | Roots & Rhizomes | Antimicrobial. | S | 15 |
| *Rumex nervosus* Vahl. | Ithrib |  | Leaves & roots | Edible plant, diabetes, skin burns, inflammatory diseases, diarrhea, wounds, typhus, rabies, skin disorders. diuretic, gonorrhoea, lung tuberculosis, leprosy, antipyretic, liver disease, hypertension, haemorrhoids, scabies, antiemetic, aphrodisiac, cough, for rabies, antirheumatic & antimigraine. | S, M | 16, 12 |
| *Rumex pictus* Forssk. | Hamsees |  | Whole plant | Sedative, relieve spasm & antimicrobial. | S | 9, 15 |
| Table S1: Cont. | | | | | | |
| **Scientific name** | **Local name** | **Family** | **Part used** | **Traditional uses** | **Region** | **Reference** |
| *Rumex steudelii* | Tabal | Polygonaceae | Whole plant | Remedy for abdominal pains due to intestinal worms. | S | 15 |
| *Rumex vesicarius* L. | Hammaad |  | Whole plant | Used for toothache. | W, M, S | 13, 14, 15 |
|  |  |  | Seeds | For the cure of dysentery. |  |  |
| *Portulaca oleracea* L. | Regla | Portulacaceae | Whole plant | Anti-inflammatory, antidiabetic and for cold. | W, M, S | 13, 14 |
| *Portulaca quadifida* L. | - |  | Stems | Treatment of scorpion sting. | - | 17 |
| *Anagallis arvensis* L. | Ayn Algat | Primulaceae | Whole plant | Used for skin rash & ulcers. | - | 20 |
| *Punica granatum* L. | Ruman | Punicaceae | Fruits | Dysentery, microbial infection, diarrhea, haemorrhage & respiratory pathogenesis. | - | 24 |
| *Clematis simensis* Fresen. | Haya^,^a | Ranunculaceae | Leaves | Relieve rheumatic pain. | S | 19 |
| *Clematis wightiana* L. | - |  | Whole plant | Cardiac depression & antimicrobial. | S | 19 |
| *Nigella sativa* L. | Habbatus sauda |  | Whole plant | Carminative, mucolytic, anti-ulcers, anti-diabetes, antidote, antiasthmatic, hypertension, hepatoprotective, antiobesity, migraine, haemorrhoid & expel rodents. | S | 20 |
| *Reseda muricate* Presl. | Danban | Resedaceae | Fruit | Menstruation tonic. | M, S | 14 |
| *Ochradenus arabicus* L. | Kardey |  | Whole plant | Hypoglycemic activity. | W, S | 13 |
| *Ochradenus baccatus* Del. | Ghorzaa |  | Whole plant | Backache, fistula & antimicrobial. | W, M | 13, 14 |
| *Zizyphus nummularia* L. | Zyzafun | Rhamnaceae | Whole plant | Antispasmodic, emollient, antitussive, anti-poison, laxative & skin eruption. | S | 20 |

| Table S1: Cont. | | | | | | |
| --- | --- | --- | --- | --- | --- | --- |
| **Scientific name** | **Local name** | **Family** | **Part used** | **Traditional uses** | **Region** | **Reference** |
| *Ziziphus spina-christi* L. | Sidr, Nubak | Rhamnaceae | Whole plant & bark | Duodenum and stomach ache, remover dandruff, weaknesses, diabetes, pharyngitis, bronchitis, anemia, diarrhea, skin infections, antipyretic, sleep irritability, liver problems, digestive disorders, antimicrobial, anti-inflammatory, antiulcer, antitumor & cardiovascular disorders | W, M, S | 13, 14 |
| *Amygdalus arabica* L. | - | Rosaceae | Whole plant | Antimicrobial. | S | 19 |
| *Cotoneaster nunnularia* | - |  | Whole plant | Purgative, antiflatulence, expectorant, appetizer, digestive, antitussive, aperient & stomachic. | M | 12 |
| *Prunus amygdalus* L. | - |  | Whole plant | As aphrodisiac. | S | 15 |
| *Rosa abyssinica* Lindley. | Aball |  | Whole plant | Antimicrobial. | S | 19 |
| *Coffea arabica* L. | Bun | Rubiaceae | Seeds | Relieve stomachache, colds, cough, antipyretic & toothache. | S | 16 |
| *Haplophylum tuberculatum* Forssk. | - | Rutaceae | Whole plant | Used in liver disease | S | 34, 39 |
|  |  |  | Leaves | Used as sedative, treatment of scorpion sting & strengthen week muscle. |  |  |
| *Ruta chalepensis* | Shathab |  | Leaves & stems | Relieve snake bite, headache, sore ears, rheumatism & wound healing. | S | 16 |
| *Salvadora persica* L. | Miswak | Salvadoraceae | Whole plant | Used as toothbrush & treatment of scorpion sting. | S | 15, 17 |
| *Dodonaea viscosa* Jacq, | Shath | Sapindaceae | Leaves | Used for treating chronic ulcers, burns & leishmaniasis. | S | 15, 40 |
| *Monotheca buxifolia* Falc. | But | Sapotaceae | Ripe barriers | General tonic. | S | 15, 21 |
| *Schweinfurthia pterosperma* | - |  | Whole plant | Antimicrobial. | S | 15 |
| Table S1: Cont. | | | | | | |
| **Scientific name** | **Local name** | **Family** | **Part used** | **Traditional uses** | **Region** | **Reference** |
| *Striga hermonthica* Del. | Doosh, Odaar | Scrophulariaceae | Flowers | Used for irritating & infected eyes. | S | 16 |
| *Verbascum bottae* Deflers. | - |  | Leaves & flowers | Antitussive, skin disease & antirheumatic. | S | 15, 25 |
| *Datura fastuosa* | Benj, Mang | Solanaceae | Roots, leaves, seeds & flowers | Antispasmodic, antiepileptic, headache, eye and ear diseases, epilepsy, madness, astringent, anthelmintic, parasiticide, narcotic, emetic, tuberculosis & antiasthmatic, antirheumatic. | M | 12 |
| *Datura innoxia* | Binj |  | Leaves & seeds | Colic gripes, carminative, antiasthmatic, antitussive, antitumor, hysteria, antirheumatiod, emmenagogue, flu, headache & anesthetic. | M, S, W | 12, 13, 15 |
| *Datura metel* L. | Binj |  | Leaves, flowers & seeds | Antiasthmatic, inflammation, antirheumatoid, earache & eye diseases. | S, M | 12, 15 |
| *Datura stramonium* L. | Binj-daturah |  | Leaves, roots, stems & seeds | Antirheumatic, antiasthma, antitussive, bronchitis, earache, hair fall & treatment of scorpion sting. | S, W | 15, 13, 17 |
| *Hyoscyamus albus* L. | - |  | Whole plant | Used as treatment for scorpion sting. | - | 17 |
| *Hyoscyamus muticus* L. | Asakran |  | Leaves, flowers & buds | Antitussive, expectorant, antiasthmatic, carminative, sedative, antispasmodic, sea sickness & toothache. | M | 12 |
| *Hyoscyamus pusillus* L. | Babekh Safaree |  | Seeds | Toothache. | S, M | 12, 15 |
| Table S1: Cont. | | | | | | |
| **Scientific name** | **Local name** | **Family** | **Part used** | **Traditional uses** | **Region** | **Reference** |
| *Lycium shawii* Roem. | Awsag | Solanaceae | Fruits | Mouth ulcers. | S, W, M | 13, 14, 15 |
| *Nicotiana rustica* L. | Al-tabag |  | Tobacco snuff | Useful in nasal polyp, nasal catarrh, headache, chronic giddiness, fainting, rheumatic swellings & skin diseases. | S | 15 |
| *Nicotiana tabacum* L. | - |  | Leaves | Used as treatment for scorpion sting. | - | 17 |
| *Physalis minima* L. | - |  | Whole plant | Used in gonorrhea & earache. | S | 15 |
| *Solanum anguivi* Lam. | - |  | Stems | Used as treatment for scorpion sting. | - | 17 |
| *Solanum careens* L. | - |  | Whole plant | Antimicrobial. | S | 15 |
| *Solanum forskalii* Dunal*.* | Nashbah |  | Whole plant | Used for treating ulcers. | W, S | 13, 15 |
| *Solanum glabratum* Dunal. | - |  | Leaves & fruits | Diuretic, for scabies, cough & hemorrhoids. | S | 15 |
| *Solanum incanum* L. | Aeen Elbagar |  | Fruits | Antimicrobial. | W, S | 13, 41 |
| *Solanum nigrum* L. | Enab-Alzeeb |  | Whole plant | Used in jaundice, antipyretic, gonorrhea, diarrhea, heart diseases, inflammation, edema, mastitis & hepatic cancer. | S | 15 |
| *Solanum surattense* Burm. f. | Bankum-Bakini |  | Whole plant | Antipyretic, antiasthmas, antitussive, in sexual diseases & to promote female fertility. | S | 34 |
| *Solenostemma argel* (Del.) Hayne. | Al-Argal |  | Leaves | Antirheumatic & antitussive. | S | 15 |
| *Withania somnifera* L. | Sem-alfaar |  | Leaves | Used in ulcers. | W, M, S | 13, 14, 17 |
|  |  |  | Whole plant | Antirheumatic, dyspepsia, appetizer & for edem. |  |  |
|  |  |  | Roots | Used as tonic for uterus of women after habitual miscarriage. |  |  |
|  |  |  | Stems | Used as treatment for scorpion sting. |  |  |
| Table S1: Cont. | | | | | | |
| **Scientific name** | **Local name** | **Family** | **Part used** | **Traditional uses** | **Region** | **Reference** |
| *Tamarix amplexicaulis* L. | - | Tamaricaceae | Whole plant & roots | For central nervous system depression, cardiac stimulation & antimicrobial. | S | 19 |
| *Tamarix aphylla* L. | Athel |  | Leaves & roots | Wound infection & stomachache. | W, M, S | 13, 14 |
| *Tamarix nilotica* Ehrenb. | Tarfaa |  | Leaves & seed^,^s oil | Used for leg varices. | M, S | 14 |
| *Gnidia somalensis* Franch. | Barha | Thymelaceae | Leaves | As emetics & purgatives. | S | 21 |
| *Grewia tenax* Forssk. | Khadar | Tiliaceae | Wood & barks | Antitussive, analgesic, central nervous system depression & antimicrobial. | M, S | 12, 15 |
| *Triumfetta flavescens* L. | - |  | Whole plant | Central nervous system stimulant & cardia stimulation. | S | 19 |
| *Typha domingensis* (Pers.) Poir. | Pardey | Typhaceae | Whole plant | Cardiac depression. | S | 19 |
| *Forsskalea tenacissima* L. | Lussaique | Urticaceae | Whole plant | Used for ulcers | M, W | 14, 13 |
| *Urtica urens* L. | Hurraikha |  | Leaves | Used for muscle pain & antirheumatic. | S | 16 |
| *Lantana petitiana* | Saaf | Verbenaceae | Leaves & roots | Used for abdominal colic. | - | 21 |
| *Phyla odiflora* L. | - |  | Leaves | Used as treatment for scorpion sting. | - | 17 |
| *Cissus rotundifolia* L. | Ghalaf | Vitaceae | Whole plant | Heated plant used to relieve backache. | S | 16 |
| *Cyphostemma ternatum* | Kum |  | Leaves & stems | Used to treating infections. | - | 21 |
| *Alpinia galangal* L. | - | Zingeberaceae | Rhizomes | Used against kidney stones. | S | 23 |
| *Alpinia officinalis* L. | - |  | Roots & rhizomes | Antirheumatic & children whooping cough. | S | 15 |
| *Curcuma longa* L*.* | Kurkum |  | Rhizomes | Skin disorders, bronchitis, antitussive & eye infections. | S | 15 |

| Table S1: Cont. | | | | | | |
| --- | --- | --- | --- | --- | --- | --- |
| **Scientific name** | **Local name** | **Family** | **Part used** | **Traditional uses** | **Region** | **Reference** |
| *Zingiber officinalis* L. | Zingibil | Zingeberaceae | Rhizomes | Hepatoprotective, clears vision, digestive, aphrodisiac, gout, antirheumatic, voice clearness, antipyretic, antiscorbutic & food condiment. | S | 20 |
| *Fagonia bruguieri* Prod. | Shika^,^a | Zygophyllaceae | Whole plant | Antipyretic, antiasthmatic, anti-emetic, dysentery, typhoid, anti-toxic, anti-tumor, blood and heart tonic & for ulcers. | W, M, S | 12, 13, 14 |
| *Fagonia indica* Burm. | Showaika |  | Whole plant | Used for smallpox & gout. | M, W, S | 12, 13, 14 |
| *Peganum harmala* L. | Harmal |  | Seeds | Sexual stimulation. | W, S | 13, 16 |
| *Tribulus terrestris* L. | Shirshir |  | Leaves | Renal colic. | W, M, S | 12, 13, 14 |
| *Zygophyllum album* L. | Retret |  | Whole plant | Antidiabetic & cardiovascular disease. | S | 15 |
| *Zygophyllum coccineum* L. | Harm |  | Whole plant | Anthelmintic. | M, S | 13, 14 |
| *Zygophyllum simplex* L. | AL-damran |  | Whole plant | Used in ophthalmic disease. | W, M, S | 12, 13, 14 |
| W= west region of KSA, S= south region of KSA, M=middle region of KSA | | | | | | |


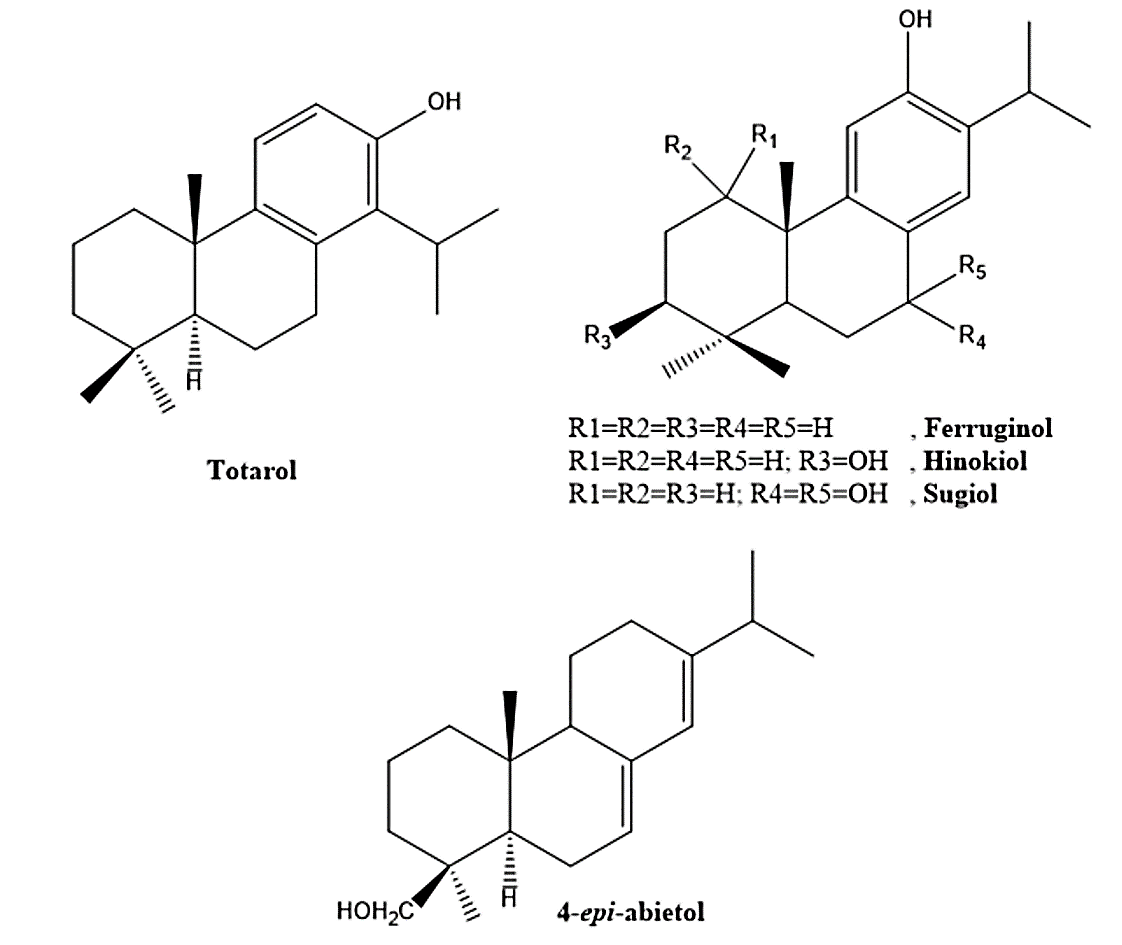


Figure S1: Structures of the major compounds isolated from *J. procera*


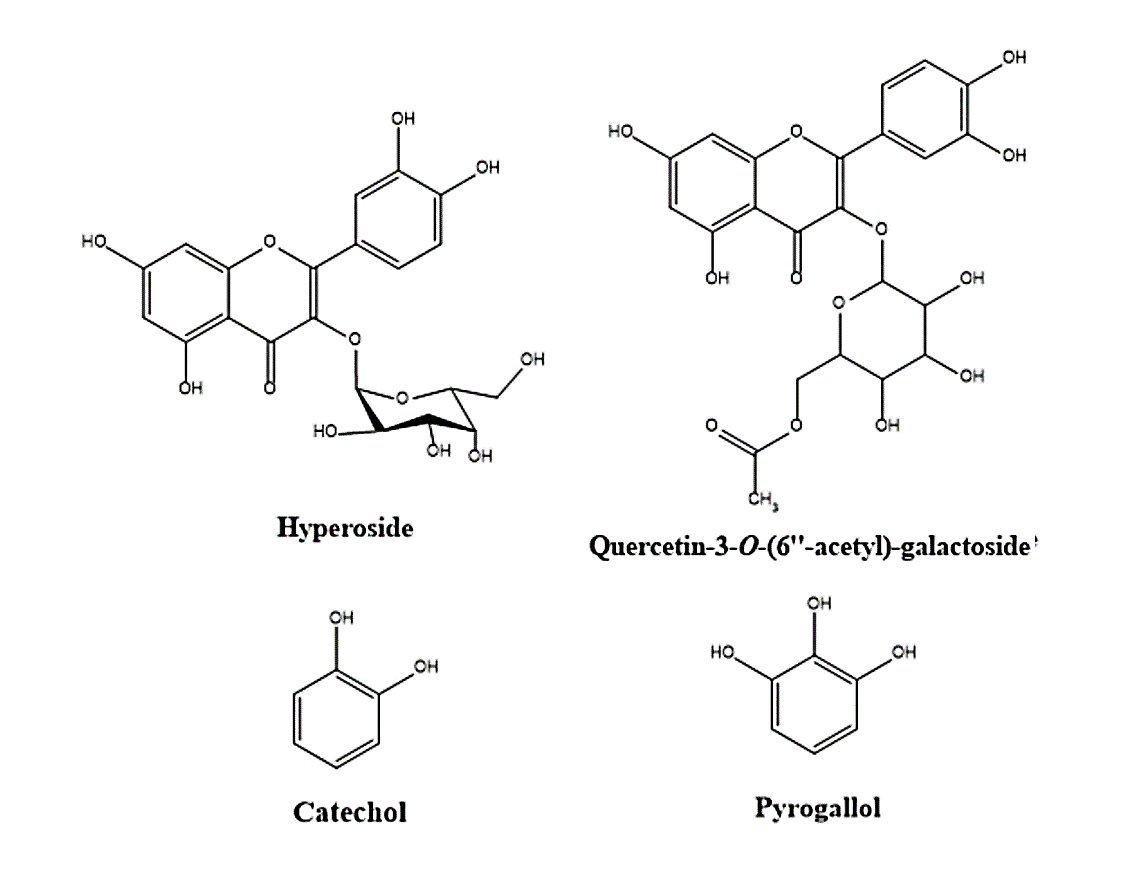


Figure S2: Structures of the major compounds isolated from *R. nervosus*


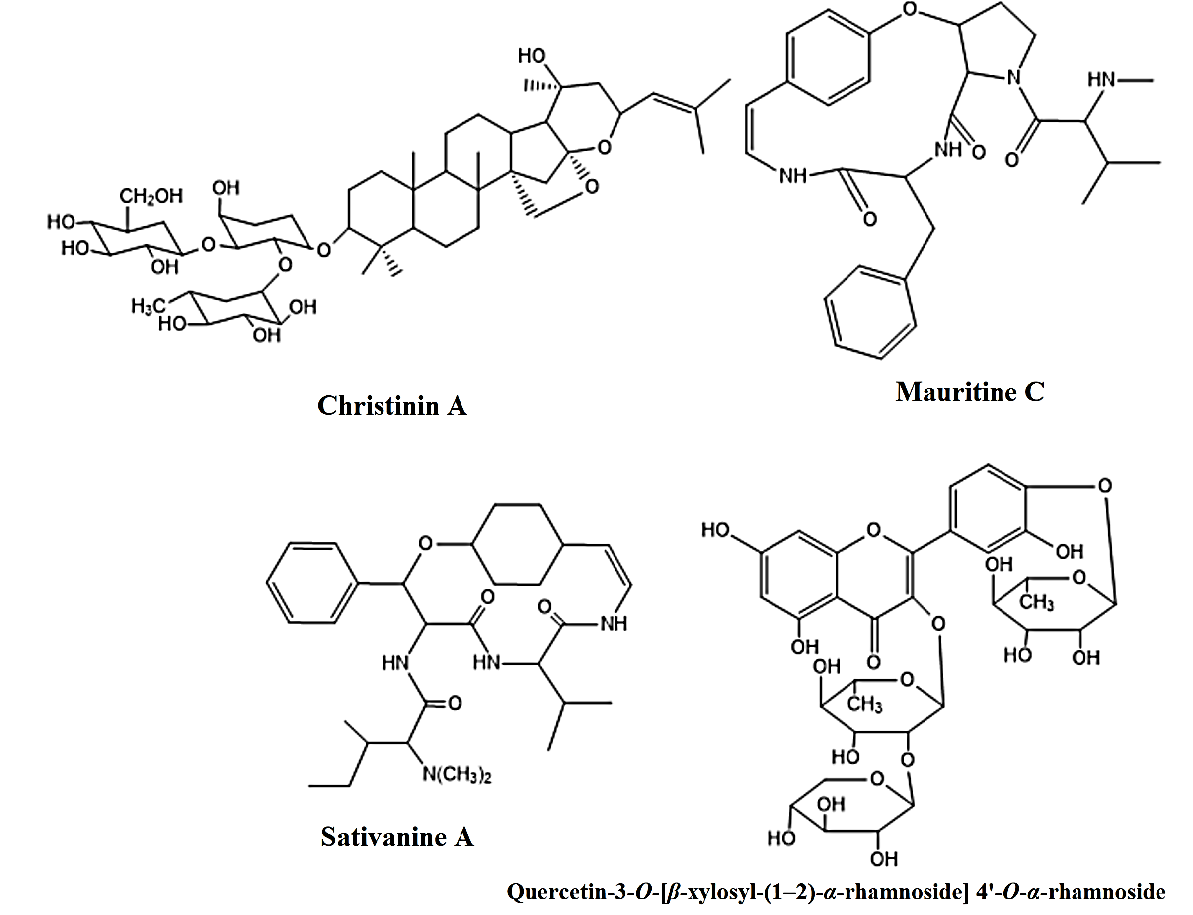


Figure S3: Structures of the major compounds isolated from *Z. spina-christi*
